# Unlocking the Potential of CuAgZr Metallic Glasses: A Comprehensive Exploration with Combinatorial Synthesis, High‐Throughput Characterization, and Machine Learning

**DOI:** 10.1002/advs.202302997

**Published:** 2023-09-23

**Authors:** Krzysztof Wieczerzak, Alexander Groetsch, Krzysztof Pajor, Manish Jain, Arnold M. Müller, Christof Vockenhuber, Jakob Schwiedrzik, Amit Sharma, Fedor F. Klimashin, Johann Michler

**Affiliations:** ^1^ Swiss Federal Laboratories for Materials Science and Technology Laboratory of Mechanics of Materials and Nanostructures Empa Feuerwerkerstrasse 39 Thun CH‐3602 Switzerland; ^2^ Department of Materials Science and Engineering University of California Irvine CA 92617 USA; ^3^ Faculty of Metals Engineering and Industrial Computer Science AGH University of Science and Technology Al. Mickiewicza 30 Krakow 30059 Poland; ^4^ School of Mechanical and Manufacturing Engineering University of New South Wales (UNSW Sydney) Kensington NSW 2052 Australia; ^5^ Laboratory of Ion Beam Physics ETH Zurich Schafmattstrasse 20 Zurich CH‐8093 Switzerland

**Keywords:** high‐throughput characterization, machine learning, material library, mechanical properties, metallic glasses

## Abstract

In this work, the CuAgZr metallic glasses (MGs) are investigated, a promising material for biomedical applications due to their high strength, corrosion resistance, and antibacterial activity. Using an integrated approach of combinatorial synthesis, high‐throughput characterization, and machine learning (ML), the mechanical properties of CuAgZr MGs are efficiently explored. The investigation find that post‐deposition oxidation in inter‐columnar regions with looser packing causes high oxygen content in Cu‐rich regions, significantly affecting the alloys' mechanical behavior. The study also reveals that nanoscale structural features greatly impact plastic yielding and flow in the alloys. ML algorithms are tested, and the multi‐layer perceptron algorithm produced satisfactory predictions for the alloys' hardness of untested alloys, providing valuable clues for future research. The work demonstrates the potential of using combinatorial synthesis, high‐throughput characterization, and ML  techniques to facilitate the development of new MGs with improved strength and economic feasibility.

## Graphical abstract

This study explores the mechanical properties of CuAgZr metallic glasses, a material with potential for biomedical applications, using combinatorial synthesis, high‐throughput characterization, and machine learning. The multi‐layer perceptron algorithm effectively predicts the hardness of untested alloys, demonstrating the potential of these techniques in developing new, economically feasible metallic glasses with improved strength.

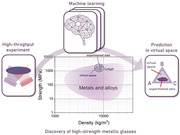

## 1 Introduction

The field of materials science has been witnessing a rapid surge in the development and discovery of novel materials, which holds the key to unlocking unprecedented technological advancements. Multicomponent materials such as MGs have garnered significant attention due to mechanical and physicochemical properties that are not observed in conventional crystalline alloys such as high hardness and strength,^[^ ^1^ ^]^ good wear resistance,^[^ ^2^ ^]^ excellent corrosion resistance,^[^ ^3^ ^]^ and high elasticity.^[^ ^4^ ^]^ MGs are typically composed of three or more elements and produced in a narrow composition range in the vicinity of deep eutectics.^[^ ^5^, ^6^ ^]^ However, the vast compositional space associated with multicomponent systems presents a significant challenge in identifying and optimizing materials with desirable properties. Consequently, there is a pressing need for innovative approaches that can accelerate the exploration of this complex landscape.

Combinatorial synthesis of material libraries (MatLib) has emerged as a powerful strategy to address this challenge. Among the various techniques employed for combinatorial synthesis, magnetron co‐sputtering has proven to be a highly effective and versatile method for depositing thin film materials with controlled compositions and structures.^[^ ^7^, ^8^, ^9^ ^]^ Magnetron co‐sputtering allows the synthesis of compositional‐focused MatLibs, where the concentration of each constituent varies across the substrate, allowing for the rapid screening and optimization of material properties. A large amount of experimental data produced by high‐throughput screening makes it possible to employ ML techniques, which have been found to be particularly useful in this endeavor,^[^ ^10^ ^]^ as they can be used to identify patterns and relationships in the data that may not be immediately apparent. Furthermore, ML models can also be used to predict the properties of new metallic glass compositions, which can greatly accelerate the discovery process.

In this work, we synthesized compositionally‐focused CuAgZr MatLib within a range of chemical compositions exhibiting high glass forming ability (GFA). Produced MGs and amorphous/crystalline composites were characterized using high‐throughput methods to determine their chemical composition, impurity levels, structure, and the effects of these factors on mechanical behavior. The generated database was employed to train a ML model, which subsequently predicted the mechanical properties of untested alloys in virtual space. These predictions were validated using a separate set of experimental data, which were not included in the training set. The objective of this work was to utilize combinatorial synthesis, high‐throughput characterization, and ML to efficiently screen the mechanical properties of CuAgZr MGs. These alloys are particularly interesting due to their unique combination of high strength, corrosion resistance, and antibacterial activity.^[^ ^11^, ^12^ ^]^ Given these properties, CuAgZr MGs show great potential as coatings for biomedical applications, where high mechanical properties are crucial for ensuring durability. Investigating the underlying mechanisms governing the mechanical behavior of CuAgZr metallic glasses is essential to identify the best candidates and optimize their properties for specific applications. Moreover, our research results enable the identification of guide alloys for scaling up and producing bulk metallic glasses, thus paving the way for technological advancements and innovation.

## 2 Results

### 2.1 Chemistry and Structure

**Figure 1:**
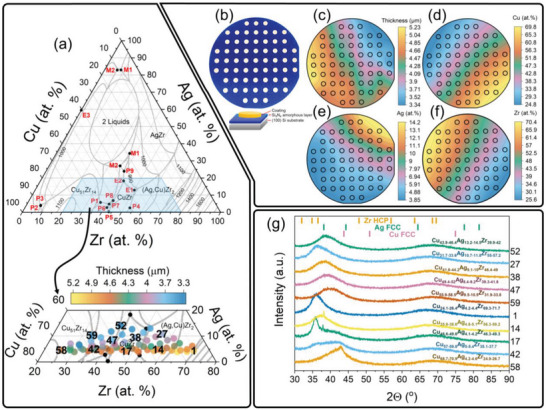
Chemical and structural analysis of the CuAgZr MatLib. (a) Calculated liquidus surface projection in the CuAgZr system via the Calculation of Phase Diagrams (CALPHAD) method, adapted from.^[^ ^15^ ^]^ E – ternary eutectic, P – ternary peritectic, M – ternary monotectic. On the slice of this ternary system thickness and compositional distribution of the as‐deposited CuAgZr MatLib, measured with XRF, is presented. (b) The appearance of the as‐deposited CuAgZr MatLib. (c)–(f) Contour plots for the thickness and elemental concentration of the investigated thin film on a wafer. (g) X‐ray diffractograms of selected regions of MatLib, marked in Figure 1a, showing amorphous or quasi‐amorphous structures. (For interpretation of the references to color in this figure legend, the reader is referred to the web version of this article).

Experimental studies have shown that MGs can be stabilized in the vicinity of deep eutectic valleys,^[^ ^13^, ^14^ ^]^ therefore to increase the chances of the amorphization of the synthesized alloys, we focused on the most reactive region of the CuAgZr system based on the liquidus surface projection, assessed by Kang and Jung^[^ ^15^ ^]^ and shown in **Figure** **1**a. The authors predicted the occurrence of the stable liquid miscibility gap in the middle of the ternary system (“2 Liquids” in Figure 1a), which is associated with the tendency to immiscibility in Ag‐Cu and Ag‐Zr binary systems. Another characteristic feature of this system is the high density of peritectic and eutectic valleys in the region with low Ag content (<25 at. %), thus, the physical vapor deposition (PVD) process was calibrated to synthesize a MatLib located in this region. The composition range of the synthesized MatLib overlaps very well with the reactive region of the CuAgZr system, i.e., where peritectic and eutectic valleys are located. Figure 1b presents a photo of the as‐deposited CuAgZr MatLib onto silicon nitride‐coated substrate in the form of 61 patches with a diameter of 5 mm, uniformly distributed on the silicon wafer. The marking of patches on the tested MatLib is shown in Figure S1 (Supporting Information). The chemical composition of each patch was determined at its center via X‐ray fluorescence (XRF) spectroscopy, with a spot size of 0.3 mm. Figure 1c–f show thickness and compositional distribution across the wafer (results are also summarized in Table S1, Supporting Information). The obtained film thickness varies across the wafer in the range between 3.34 and 5.23 µm, which results from the different deposition rates of the sputtered elements. Regions with a high concentration of a given element were located in close proximity to the corresponding magnetron. A steep concentration gradient can be observed in these areas. As the distance from the magnetron increases, the concentration gradient smoothly turns into an almost flat distribution. Figure 1g shows X‐ray diffraction (XRD) results of selected patches, marked in Figure 1a. It should be noted that the crystallographic information was obtained from the entire patch with a diameter of 5 mm, so the gradients of each element's content were marked above each diffractogram. The method employed to determine the gradient of the chemical composition started with the use of XRF spectroscopy. This was utilized to identify both the center position of each patch and the content of each element. Subsequently, a bivariate cubic function was used to define chemical composition surfaces, representing the elemental content as the function of the x, y position. Leveraging these surfaces, the gradient for each element within the patches was then calculated. This methodology offered a systematic approach for assessing the direction of elemental content change within each patch of the investigated MatLib. It's important to note that the fit of the function, represented by the R^2^ value, was greater than 0.99 in every case, indicating a high degree of accuracy in the model.

Broad diffraction peaks confirm amorphous or quasi‐amorphous structure of the investigated alloys, which proves that the alloys located near the peritectic and eutectic valleys have good GFA. For Cu_37.1_Ag_4.8_Zr_58.1_ (P14) and Cu_48.1_Ag_4.2_Zr_47.8_ (P17) alloys, sharp and low‐intensity peaks can be observed that correspond to the Ag‐rich face‐centered cubic (FCC) phase and suggest segregation of Ag, most likely into the inter‐columnar regions. This can be deduced from the work of Huszar et. al.^[^ ^16^ ^]^ who studied a thin film of the Cu_42_Ag_5_Zr5_3_ alloy and observed a clearly increased concentration of Ag in the inter‐columnar regions. The asymmetrical and wide shape of the main peaks for the Cu_58.5_Ag_5_Zr_36.5_ (P42) alloy indicates the presence of at least two phases with an amorphous or nanocrystalline structure for this alloy.

### 2.2 Oxygen Contamination

**Figure 2:**
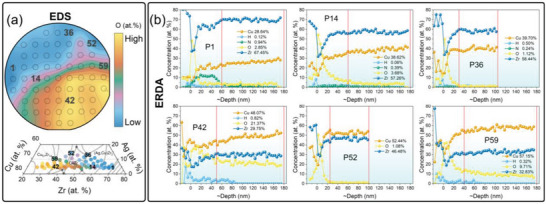
A semiquantitative oxygen analysis performed on the CuAgZr MatLib using EDS (a) and a normalized quantitative ERDA depth profiles analysis of selected alloys (b). The regions selected for the ERDA analysis are marked in Figure 2a. Note that the depth was estimated approximately based on the average theoretical alloy density determined from the Rutherford backscattering spectrometry (RBS) measurements. Values quoted to the right of the profiles represent average concentration in the depth range marked by red lines. (For interpretation of the references to color in this figure legend, the reader is referred to the web version of this article).

**Figure 3:**
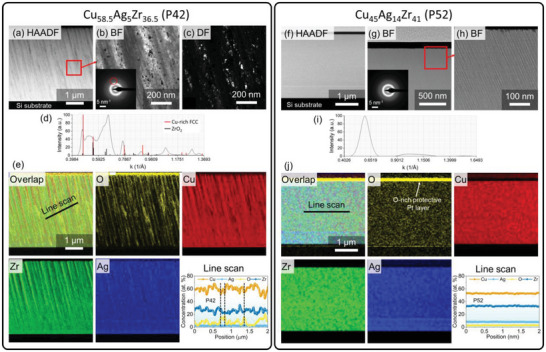
TEM analysis of the Cu_58.5_Ag_5_Zr_36.5_ (P42) and Cu_45_Ag_14_Zr_41_ (P52) alloys. (a)–(c) TEM images displaying the columnar structure of the Cu_58.5_Ag_5_Zr_36.5_ alloy. SAED patterns (inset in (b)) of the Cu_58.5_Ag_5_Zr_36.5_ alloy showing diffuse pattern corresponding to amorphous structure, decorated by spots corresponding to crystalline regions. The part of the diffraction pattern marked by a red circle was used for DF imaging (c) of the same region as shown in (b), where it can be seen that the crystalline phases are located in the regions between the amorphous columns. (d) Intensity profile taken from the SAED pattern (b) with crystalline phase's identification. (e) TEM‐EDS maps and line scan showing enrichment in oxygen of the inter‐columnar regions. (f)–(h) TEM images displaying columnar structure of the Cu_45_Ag_14_Zr_41_ alloy showing clearly narrower columns (6–8 nm) compared to the Cu_58.5_Ag_5_Zr_36.5_ alloy (70–100 nm). (i) Intensity profile taken from the SAED pattern (g) indicating fully amorphous structure. (j) TEM‐EDS maps and line scan showing homogeneous distribution of elements within the alloy. HAADF – high‐angle annular dark field, BF – bright‐field, DF – dark‐field. (For interpretation of the references to color in this figure legend, the reader is referred to the web version of this article).

The mechanical properties of metals strongly depend on the amount of impurities,^[^ ^17^ ^]^ therefore detailed research was carried out on their content in the tested CuAgZr MatLib. Due to the fact that a wide range of chemical compositions have been produced, large variations in the content of contaminants such as oxygen can be expected. **Figure** **2** displays the results of a semiquantitative oxygen analysis carried out in the center of each patch via energy dispersive X‐ray spectroscopy (EDS) (results are also summarized in Table S1, Supporting Information) and a quantitative oxygen analysis of selected patches via elastic recoil detection analysis (ERDA). EDS measurements (Figure 2a) show a clear trend in oxygen distribution, i.e., its concentration increases towards the Cu‐rich region. Figure 2b presents quantitative ERDA depth profiles of selected samples, marked in Figure 2a. The ERDA results are in good agreement with EDS measurements. In the low oxygen regions according to EDS, i.e., Cu_25.4_Ag_4.3_Zr_70.3_ (P1), Cu_35.7_Ag_13_Zr_51.3_ (P36), Cu_45_Ag_14_Zr_41_ (P52), a relatively low oxygen content was found by the ERDA method, i.e., 2.85, 1.12 and 1.08 at. %, respectively. On the other hand, in the oxygen‐rich region according to the EDS, a surprisingly high oxygen content of 21.37 at. % for the Cu_58.5_Ag_5_Zr_36.5_ alloy (P42) was found by ERDA. As the origin of oxygen in the tested MatLib is unclear, a Transmission Electron Microscopy (TEM) experiment was conducted to analyze its distribution and solve this puzzle. **Figure** **3** shows the results of TEM analysis on an alloy with high oxygen content Cu_58.5_Ag_5_Zr_36.5_ (P42) and low oxygen content Cu_45_Ag_14_Zr_41_ (P52). The Cu_58.5_Ag_5_Zr_36.5_ (P42) alloy exhibits a columnar structure with a width of columns in the range of 70 – 100 nm (Figure 3a–c). Selected area electron diffraction (SAED) pattern revealed quasi‐amorphous phase composition. Dark‐field (DF) imaging exposed the location of crystalline phases in the inter‐columnar regions (Figure 3c). The crystalline phases, i.e., Cu‐rich FCC phase and ZrO_2_, were identified through indexing of intensity profile (Figure 3d) taken from the SAED pattern. Figure 3e shows the distribution of elements in the Cu_58.5_Ag_5_Zr_36.5_ (P42) alloy. There is a clear enrichment of the inter‐columnar regions with oxygen. Line scan analysis revealed that the oxygen‐enriched areas are simultaneously Cu‐depleted as shown by the dashed lines in Figure 3e, further indicating the formation of ZrO_2_. The structure of the Cu_45_Ag_14_Zr_41_ (P52) alloy is clearly more homogeneous (Figure 3f–h). Nevertheless, at high magnification, a fibrous structure can be observed with column widths in the range of 6–8 nm. SAED confirm the fully amorphous structure of the investigated alloy (Figure 3g,i) which agrees with the XRD experiment (Figure 1g), while TEM‐EDS analysis shows a uniform distribution of elements in the alloy (Figure 3j). The presence of a columnar structure in a coating is in line with Thornton's structural zone model of coating growth.^[^ ^18^ ^]^ This model suggests that if the ratio of substrate temperature (*T*) to the melting point of the material (*T_m_*) is less than 0.3, then columnar growth with voided boundaries can be expected. On the other hand, if the *T/T_m_* ratio is between 0.3 and 0.5, the formation of dense inter‐columnar boundaries is expected. In the case of compositionally‐focused MatLib different film morphologies can be expected due to variations in *T_m_* as a function of chemical composition (Figure 2a), even if *T* remains constant during synthesis.

### 2.3 Mechanical Performance

**Figure 4:**
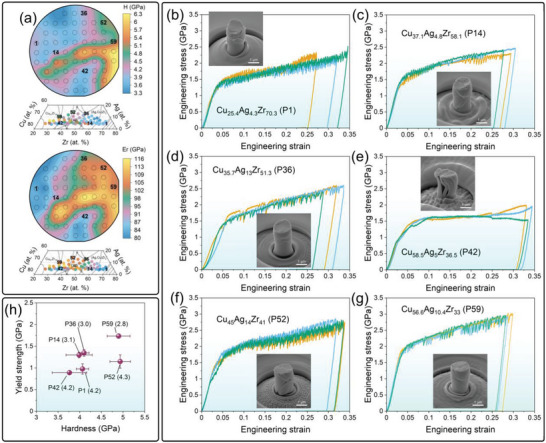
Mechanical properties of CuAgZr MatLib. (a) Results of hardness (H) and reduced Young's modulus (Er) mapping. (b)–(g) In situ compression experiments. Compressive engineering stress–strain curves with exemplary SEM images of micropillars after deformation in selected regions of the CuAgZr materials library, marked in Figure 4a. (h) Yield strength versus hardness. The values next to the data points represent the hardness to yield strength (H/σ_y_) ratio. (For interpretation of the references to color in this figure legend, the reader is referred to the web version of this article).

The effect of chemical composition on hardness (*H*) and reduced elastic modulus (*Er*) is shown in **Figure** **4**a (results are also summarized in Table S1, Supporting Information). Both *H* and *E_r_* distribution show a non‐obvious pattern as they do not follow a chemical composition gradient (Figure 1). Surprisingly, the lowest *H* and *E_r_* are found in Zr‐rich regions, while the highest values of the above properties were identified in Cu‐rich regions. This surprise is due to the fact that Zr has the highest melting point among the elements in the CuAgZr system, which is directly related to bond energy. It is worth noting that alloys with similar *H* may differ in *E_r_* by up to 20 GPa. No clear correlation between the mechanical properties and the oxygen content (Figure 2) was found based on their distribution.

Figure 4b–g show results of micropillar compression in selected alloys from the CuAgZr system, marked in Figure 4a, with exemplary scanning electron microscope (SEM) images of micropillars after deformation. The micropillars were produced in close proximity, so the differences in the content of individual elements are less than 0.001 at. %, which is manifested by a very good reproducibility of the results and a small standard deviation of yield strength σ_y_ (results are summarized in Table S2, Supporting Information). The σ_y_ of the investigated alloys is in the range from 0.89 GPa for Cu_58.5_Ag_5_Zr_36.5_ (P42) alloy to 1.74 GPa for Cu_56.5_Ag_10.4_Zr_33_ (P59) alloy. Moreover, the serrated flow behavior from the load‐displacement response can be seen for most alloys except the high‐oxygen Cu_58.5_Ag_5_Zr_36.5_ (P42) alloy. In the case of Cu_37.1_Ag_4.8_Zr_58.1_ (P14), Cu_35.7_Ag_13_Zr_51.3_ (P36), and Cu_56.5_Ag_10.4_Zr_33_ (P59) alloys, the deformation character is mixed, i.e., serrated alternates with homogeneous (monotonic segments on the stress‐strain curve after exceeding the yield point). This demonstrates the profound influence of nanoscale structural features on plastic yielding and flow in the tested alloys. SEM images reveal the occurrence of multiple shear bands, which indicate that one shear band was not sufficient to dissipate the applied energy. Formation of a single shear band causes stress unloading or strain relaxation in their vicinity. Therefore, new shear bands appear at a certain characteristic spacing. It should be noted here that the multiplication phenomenon of shear bands does not depend solely on the chemical composition,^[^ ^19^ ^]^ but may also depend on the sample geometry,^[^ ^20^ ^]^ loading mode^[^ ^21^ ^]^ and atomic topological aspects (free volume, defects, etc.).^[^ ^22^ ^]^ Regarding the post mortem nature of the micropillar shape, the oxygen‐rich Cu_58.5_Ag_5_Zr_36.5_ (P42) alloy is clearly different from the others. The alloy is characterized by a columnar structure and contains a certain fraction of crystalline phases, i.e., Cu‐rich FCC and ZrO_2_ phases (Figure 3). In the highly deformed micropillar the columns underwent splitting and a high degree of bending. Figure 4h shows correlation between Yield strength and hardness. The Cu_37.1_Ag_4.8_Zr_58.1_ (P14), Cu_35.7_Ag_13_Zr_51.3_ (P36), and Cu_56.5_Ag_10.4_Zr_33_ (P59) alloys that had mixed character of deformation follow the classical relationship H≈3σ_y_, proposed by Ashby and Jones, and Tabor.^[^ ^23^ ^]^ Other alloys have a relationship H>3σ_y_ typical for glasses that display brittle behavior even under compression.^[^ ^24^, ^25^ ^]^ This group includes both low‐oxygen (Cu_25.4_Ag_4.3_Zr_70.3_ (P1) and Cu_56.5_Ag_10.4_Zr_33_ (P59)) alloys, and high‐oxygen (Cu_58.5_Ag_5_Zr_36.5_ (P42)) alloy, so the main alloying elements are responsible for these characteristics.

### 2.4 Prediction of the Hardness of Untested Alloys in Virtual Space

The experimental dataset on hardness/strength provided in this work obtained using high‐throughput nanoindentation enables the application of machine learning methods to identify relevant patterns and correlations between input variables such as topological, chemical, electronic structure, mechanical descriptors, and desired properties (e.g., hardness or strength). A predictive model developed on this basis can subsequently be used to forecast the properties of alloys that extend beyond the compositional space studied in this work.

**Figure 5:**
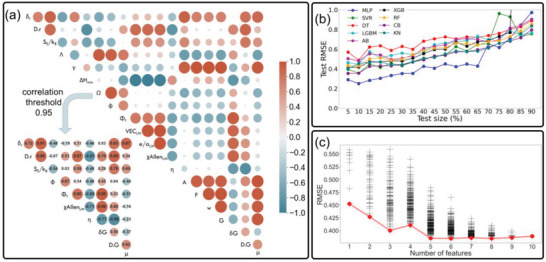
Selection of the features built based on domain knowledge. (a) The Pearson correlation map of the twenty initial features. The diameter and color of the circles represent the strength of the correlation. After setting the threshold at 0.95, the analysis identified features with high correlation, and one feature from each correlated pair was dropped leaving ten features. (b) Test RMSE for various machine learning models as a function of test set size. The plot illustrates the performance of each model trained using ten features, selected based on correlation analysis, in terms of prediction error on unseen data. Lower RMSE values indicate better model performance. The algorithms used in the study included K‐Nearest Neighbors Regression (KN),^[^ ^27^ ^]^ Support Vector Regression (SVR),^[^ ^28^ ^]^ Multilayer Perceptron Regression (MLP),^[^ ^29^ ^]^ XGBoost Regression (XGB),^[^ ^30^ ^]^ Decision Tree Regression (DT),^[^ ^31^ ^]^ Random Forest Regression (RF),^[^ ^32^ ^]^ AdaBoost Regression (AB),^[^ ^33^ ^]^ LightGBM Regression (LGBM),^[^ ^34^ ^]^ and CatBoost Regression (CB).^[^ ^35^ ^]^ (c) The relationship between the number of features utilized in an MLP model and the RMSE of the model's predictions. Each point (+) represents a different feature combination. The red line denotes the minimum RMSE for each number of features. (For interpretation of the references to color in this figure legend, the reader is referred to the web version of this article).

**Figure** **5**a displays the Pearson correlation coefficient map between different features. Those with a correlation coefficient greater than 0.95 were considered highly correlated, which implies that these variables encompass similar or identical information. Our choice of 0.95 as the threshold was carefully considered: it was a balance between maintaining enough features for a comprehensive analysis and eliminating redundant or closely linked variables. This threshold allowed us to retain ten distinct features (as seen in the bottom‐left corner of Figure 5a) from the originally highly‐correlated pool. The reason for this selection was to effectively reduce dimensionality, which consequently decreases the model's complexity, simplifies its interpretation, and reduces the risk of overfitting. While a correlation coefficient threshold of 0.8 is also generally considered to indicate a very strong correlation according to Evans's guidelines,^[^ ^26^ ^]^ adopting this threshold in our analysis would have significantly reduced the number of features left for examination. Therefore, the 0.95 threshold was selected as a way to ensure a reasonable balance between retaining significant features and minimizing redundancy.

Figure 5b illustrates the performance of various machine learning models in terms of their best root mean square error (RMSE) over a wide range of test set sizes from 5% to 90% to provide a more complete understanding of the performance and capabilities of the machine learning models under study. For a more comprehensive overview, we have additionally included in Figure S2 (Supporting Information) on the training RMSE, and overfitting/underfitting (test RMSE – training RMSE) curves for different machine learning models. From Figure 5b it can be observed that the performance of the models varies across different test set sizes. For several models, e.g., Multilayer Perceptron Regression (MLP), Support Vector Regression (SVR), and Decision Tree Regression (DT), increasing the test size from 5% to 10% results in a decrease in RMSE, a phenomenon largely attributable to overfitting at smaller test size (Figure S2, Supporting Information). As the test size expands, the RMSE decreases for the majority of models. After exceeding a specific threshold of ≈10% for most ML models, the RMSE begins to rise again. This pattern emerges because, with a larger test set, the model has access to less training information. In other words, this might suggest that the model is underfitting or that the model is too simple to generalize well on new data. In this case, the model may struggle to predict data that was not part of the training set, resulting in a large error on the test data. It is important to note that a lower test RMSE signifies a better fit of the model to the data, as it represents the average deviation between the predicted values and the true values. The MLP model not only outperforms the other models in the test size range between 5% and 65%, but it also maintains a low level of overfitting, especially in the range below 25% in the test size (Figure S2, Supporting Information). Therefore, due to its superior performance and ability to generalize well, it was chosen for further optimization and for predicting hardness in the virtual space. Figure 5c displays the relationship between the number of features used in the fine‐tuned MLP model and the corresponding RMSE for various feature combinations. Each black cross (+) on the graph represents a unique combination of features used in the model, and its position on the y‐axis indicates the RMSE value obtained for that specific combination. The purpose of this analysis is to identify the optimal feature set that yields the lowest RMSE and, consequently, the best model performance. The best‐performing feature sets for each number of features are summarized in Table S3 (Supporting Information). With the increase in the number of features, there is an overall downward trend in RMSE, which indicates an improvement in model performance. Nevertheless, 5 features were selected for the final model, i.e., *Φ_f_, χAllen_LM_, η, D.G, µ*, as further addition of features does not significantly reduce the RMSE.

**Figure 6:**
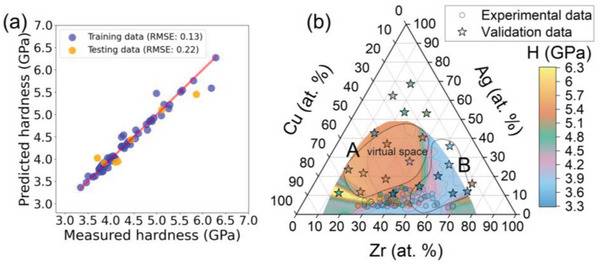
a) Scatterplot of measured hardness versus predicted hardness using the fine‐tuned and trained MLP model. b) The predicted hardness of unexplored alloys in virtual space. The experimental data marked with circles on the figure were used to train the MLP model. The validation data, marked with stars, were used to evaluate the predictions of the MLP model, as this model has never seen these data before. This approach ensures an unbiased evaluation of the model's predictive performance. (For interpretation of the references to color in this figure legend, the reader is referred to the web version of this article).

To predict the hardness, an optimized MLP model with an architecture containing two hidden layers with 8 and 6 neurons, respectively, was used. **Figure** **6**a shows a scatterplot comparing the predicted hardness values with the measured hardness values for both the training and test sets (chosen randomly and representing 10% of the data) and providing a visual representation of the model's performance and its ability to generalize to new data. The red diagonal line indicates perfect agreement between the measured and predicted values. The model shows good predictive power for both training and testing sets, offering hardness RMSE of 0.13 and 0.22 GPa, respectively. This shows the model's potential applicability in predicting the hardness of CuAgZr metallic glasses. Figure 6b shows the predicted hardness of unexplored alloys in virtual space. The virtual space was constructed based on the single criterion *δ_r_* ×100 > 8. The *δ_r_* criterion allows predicting alloys with increased propensity to form metallic glasses that was empirically confirmed.^[^ ^36^, ^37^ ^]^ To verify the effectiveness of this criterion, a new validation MatLib consisting of 21 patches was created. The chemical composition of these patches is presented in Table S4 (Supporting Information). The calculated chemical composition gradient and X‐ray diffractograms are presented in Figure S3 (Supporting Information). It was found that the criterion *δ_r_* ×100 > 8 is very effective in predicting the location of amorphous regions. Upon comparing the experimental data with the predictions made by the MLP model, it is evident that the model effectively captures the trend of hardness changes concerning the chemical composition. This is particularly noteworthy given the complex hardness distribution of the original data. In Figure 6b, the experimental data used to train the model and the validation data from the new MatLib consisting of 21 patches are also marked. The hardness of these patches is presented in Table S4 (Supporting Information). Despite the large distance of chemical compositions at the edges of the virtual space from the convex hull of the experimental data used to train the algorithm, the predictions are very good. Comparing the hardness in the validation set, it can be seen that the model accurately predicted the location of alloys with high hardness (area A in Figure 6b) and alloys with low hardness (area B in Figure 6b). The presence of outliers can be ascribed to the differences in synthesis parameters between the MatLib used for training and the one used for validation of machine learning predictions. Additionally, the relatively small size of the training dataset could also contribute to these discrepancies. Contaminations may be an additional factor. It should be recalled here that the features used to train the MLP algorithm were calculated based on XRF data, in which oxygen content is not included. This is a weakness of these features, as it increases the likelihood of larger deviations between prediction and experiment for alloys with high oxygen content. Nevertheless, the predictions in virtual space can serve as a valuable guide in further exploration of this and other similar systems. Issues related to impurities and mechanical properties of CuAgZr alloys will be discussed in more detail in the following sections.

## 3 Discussion

### 3.1 Preferential Oxidation

A large amount of oxygen, reaching up to 21 at. % in some alloys (Figure 2), cannot be overlooked from the point of view of mechanical properties. It is therefore crucial to understand the origin of oxygen impurities in the investigated alloy. From the point of view of the magnetron sputtering process, an important parameter affecting the amount of impurities in the deposited materials is the base pressure and the related mean free path of molecules in gas, and the time needed for a single monolayer formation.

The mean free path, *λ* (given in meters), which is defined as the average distance a particle travels between successive collisions with other particles, for air at room temperature (23 °C) can be calculated via following approximation (assuming that all the molecules that compose the gas have the same diameter):^[^ ^38^ ^]^

(1) $$\lambda =\frac{6.5\times 10^{-5}}{p}$$

where *p* is the pressure in mbar. Considering the partial pressure of oxygen (assuming the chamber's atmosphere consists of 21% oxygen) during the deposition process, the mean free path for oxygen molecules is calculated to be 516 m (at the base pressure of 6 × 10^−7^ mbar measured prior to deposition). This value is more than three orders of magnitude larger than the process chamber's greatest dimension, which is 0.42 m. The time *t*_m_ (given in seconds) to form a monolayer can be determined by:^[^ ^38^ ^]^

(2) $$t_{m}=\frac{2.49\times 10^{-6}}{p}$$

Hence, under the process vacuum conditions (base pressure), the time to form a single monolayer of oxygen molecules is 19.8 s (0.05 monolayers/s). This expression is valid when the sticking coefficient, i.e., the ratio of the number of molecules that are adsorbed on a surface for a finite period of time to the number of molecules striking the surface, is unity.^[^ ^38^ ^]^ Even taking into account the fact that the sticking probability of oxygen molecules, is ≈0.1 or less,^[^ ^39^ ^]^ the results of these considerations can be used to understand the presence of oxygen in deposited films assuming that it comes mainly from the residual gasses and other potential sources of oxygen such as targets, leaks in the gas line, etc. are negligible. The atomic diameters of Cu, Ag, and Zr are 0.2556, 0.28894, and 0.3205 nm.^[^ ^40^ ^]^ In scenarios where only pure elements are deposited, for an average deposition rate of 12 nm min^−1^, the rate of monolayer formation is 0.78, 0.69, and 0.62 monolayers/s for Cu, Ag, and Zr, respectively. This would translate to the maximum oxygen content in the films at the level of 6.5, 7.3, and 8.1 at.% for Cu, Ag, and Zr films, respectively. It should be noted that high‐purity argon was introduced during the sputtering process to achieve the desired working pressure of 5 × 10^−3^ mbar enabling high deposition rates. This resulted in a mean free path of Ar atoms of 1.6 cm and consequently multiple collisions in the process chamber and should certainly reduce the oxygen content by pushing it out of the chamber. Also, considering the fact that the sticking coefficient is significantly less than unity, as mentioned before, it should be expected that the oxygen content in the deposited films will be significantly lower (in the sub‐atomic % range). It is therefore highly unlikely that the high oxygen content in copper‐rich alloys (Figure 2) originates from residual gases.

This comprehensive study on the CuAgZr MatLib illuminates the complex relationship between alloy composition and oxygen contamination. Techniques such as EDS, ERDA, and TEM unveiled a clear increase in oxygen concentration toward the copper‐rich regions, peaking at ≈21 at.%. Despite initial assumptions that the cause of the high oxygen content is residual gases from magnetron sputtering, this was largely ruled out based on calculations of the rate of oxygen monolayer formation under deposition process conditions discussed in the previous paragraph. Unexpectedly, copper‐rich alloys exhibited high oxygen content inconsistent with thermodynamic predictions based on oxide formation energies. Considering that the formation energy at 0 K of the most stable oxides, which is −0.943 eV/atom for CuO, −0.445 eV/atom AgO, and −3.81 eV/atom for ZrO_2_,^[^ ^41^ ^]^ one would expect Zr‐rich regions to attract more oxygen than others. Of course, apart from possible changes in the energy of formation of oxides on the surface in multi‐component systems as a result of redistribution of charges and changes in chemical properties when Cu, Ag, and Zr atoms are brought into contact with each other. This observation asserts that the oxygen content is not necessarily governed by an element's affinity for oxygen, but rather hinges on the microstructural features of the alloys, notably the inter‐columnar regions typified by looser packing. Oxygen diffusion within these regions led to the post‐deposition emergence of crystalline phases such as ZrO_2_. The inter‐columnar regions should therefore be characterized by high adsorption energy which is in line with the work of Baran et al.,^[^ ^42^ ^]^ who conducted a study using first‐principles to explain the observed limiting thickness of oxide films formed on aluminum during oxidizing conditions. According to the authors, a clean surface has a very high adsorption energy for oxygen molecules, which rapidly decreases with the increase in oxide thickness. This suggests that the oxidation process is a subsequent event that takes place after the sample is withdrawn from the PVD chamber and exposed to air. Consequently, composite synthesis occurs, characterized by amorphous columns intermingled with crystalline phases (e.g., Cu‐rich FCC phase, and ZrO_2_ in the Cu_58.5_Ag_5_Zr_36.5_ (P42) alloy). This illustrates that chemical composition, process conditions, and atomic mobility within a system profoundly affect the oxygen content in the alloys studied. This also leads to the conclusion that the fraction of inter‐columnar areas with lower packing increases with an increase in the copper content of the CuAgZr system fragment under study.

### 3.2 Effect of Chemistry on Mechanical Properties

**Figure 7:**
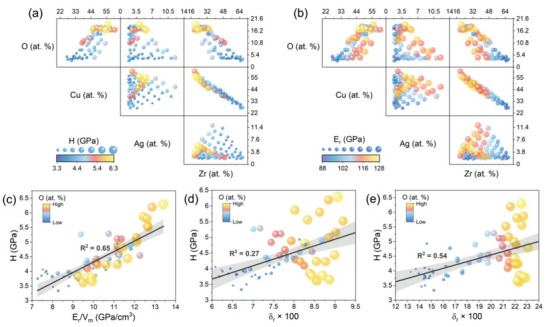
(a) and (b) correlation between the oxygen content and the content of other elements and hardness and reduced modulus, respectively. (a) and (b) The size and color of the bubbles correspond to H and E_r_ values facilitating the identification of areas with high H and E_r_ in the composition space. (c) Hardness versus normalized reduced modulus, V_m_ – molar volume, calculated based on XRF data. (d) and (e) hardness versus atomic size mismatch δ_r_, calculated based on XRF (without oxygen) and EDS (with oxygen) data, respectively. The size and color of the bubbles correspond to the oxygen content determined by the EDS. (For interpretation of the references to color in this figure legend, the reader is referred to the web version of this article).

The plastic deformation of metallic glasses at low temperatures is characterized by inhomogeneous spatial and temporal changes, carried out by localized shear bands. There have been several theories developed to explain the heterogeneous plasticity of metallic glasses, which are mainly based on two atomic‐scale mechanisms: deformation‐induced dilatation or free volume and cooperative shearing of atomic clusters called shear transformation zones (STZs).^[^ ^43^, ^44^ ^]^ STZs, small clusters of randomly close‐packed atoms, are the basic unit of plasticity and reorganize in response to applied shear stress.^[^ ^45^ ^]^ The strength of metallic glasses is influenced by a variety of factors, including the composition of the material, the cooling rate during processing, and the presence of defects or impurities.^[^ ^46^ ^]^ Combinatorial synthesis using the PVD method has the advantage of providing nearly identical processing conditions for alloys with different chemical compositions. As a result, differences in the properties of the alloys depend solely on their chemical composition, defects, and impurities. **Figure** **7**a,b show the correlation between the oxygen content and the content of other elements and mechanical properties. As mentioned earlier, high oxygen content is characteristic of copper‐rich alloys. Simultaneously, as the content of zirconium and silver in the alloy increases, the amount of oxygen decreases. This relationship is most likely related to the film growth mechanism and the number of defects that allow post‐deposition oxidation, as discussed in Section 4.1. The strength of crystalline metals is primarily influenced by the motion of dislocations, which can be assessed by considering factors like Peierls‐Nabarro forces, grain sizes, etc. In contrast, the strength of metallic glasses has been observed to be strongly linked to the physical and chemical characteristics of their constituent elements. The atomic configurations and chemical bonding forces of metallic glasses are believed to be the fundamental sources of their strength.^[^ ^47^, ^48^ ^]^ From a chemical composition point of view, an increased oxygen content would be expected to lead to improved strength due to a greater proportion of ionic bonds between metal and oxygen atoms and/or strengthening with oxides, e.g. ZrO_2_. However, the results presented in Figure 7a,b do not support this assumption, as in alloys with a high oxygen content, both very high and very low *H* and *E_r_* were reported. This can be explained by the presence in some alloys of a large fraction of regions with looser packing (inter‐columnar regions), which causes their weakening (e.g., Figure 3a–c and Figure 4e). An additional factor blurring the observed trends is the presence of crystalline regions in the case of some alloys, which can significantly affect the initiation and dominant mechanisms of plastic deformation. A good example is Cu_58.5_Ag_5_Zr_36.5_ (P42) alloy, in which the presence of a certain fraction of crystalline phases was identified (Figure 2 – Cu‐rich FCC and ZrO_2_ phases) and in which no serrated flow was observed during the micropillar compression experiment, which distinguishes it from the other alloys tested in this experiment (Figure 5). Figure 7c shows the relationship between *H* and *E*_r_. Reduced modulus here has been normalized by molar volume following Yang et al.^[^ ^48^ ^]^ The figure also includes the oxygen content in the tested alloys. Yang et al.^[^ ^48^ ^]^ observed that the normalization of the glass transition (*T_g_*) temperature using molar volume allows to emphasize the strong linear relationship with the strength of metallic glasses. This suggests that increasing the *T_g_* and decreasing the molar volume results in an increase in the strength of the metallic glass. The *T_g_* of the alloys tested in this work has not been measured and is unknown, however, the *T_g_* is strongly correlated with elastic modulus.^[^ ^49^ ^]^ Indeed, a reasonable correlation has been observed between *H* and *E*_r_ */V*_m_, which should come as no surprise as *E*_r_ reflects the bonding strength among the closest neighboring atoms in metallic glasses. Even differences in oxygen content between alloys did not cause significant deviations from this trend. In the multicomponent system atoms of different sizes can introduce some uncertainty about their position in space, which affects the number of bonds between individual atoms and, consequently, macroscopic properties. An interesting observation is that when analyzing the best set of features (Figure 5c), lattice distortion energy *µ* appeared in each set, starting with one feature (Table S3, Supporting Information). This suggests that the energy required to accommodate the differences in atomic size and structure when mixing different elements in an alloy may be correlated with the mechanical behavior of metallic glasses. Since atomic size mismatch *δ_r_* is a significant contributor to lattice distortion energy (Table S4, Supporting Information), it has been analyzed in detail in Figure 7d–e, which show *H* versus *δ_r_*. The *δ_r_* parameter was calculated using XRF and EDS data (Table S1, Supporting Information), respectively. The atomic radii were taken from Senkov and Miracle,^[^ ^40^ ^]^ where they have been critically assessed and are widely used in the field of MGs. Several interesting trends can be observed. Considering only the major alloying elements, it can be seen that an increase in atomic mismatch correlates with an increase in oxygen content (Figure 7d). On the other hand, if oxygen is included in the calculations of the lattice mismatch, it can be seen that it significantly increases it (Figure 7e). There is a linear relationship between hardness and atomic size mismatch especially for *δ_r_ ×*100 *<* ≈20, i.e., hardness increases with increasing *δ_r_* (Figure 7e). For values of *δ_r_ ×*100 > ≈20, i.e., for alloys with a high oxygen content, a significant scatter in the hardness values is observed. This suggests that the strongest alloys are those that can absorb large amounts of oxygen without having loosely packed regions causing them to weaken, such as inter‐columnar regions as in the case of the Cu_58.5_Ag_5_Zr_36.5_ (P42) alloy (Figure 3a–c and Figure 4e).

### 3.3 Accelerating Discovery of High‐Strength Metallic Glasses

**Figure 8:**
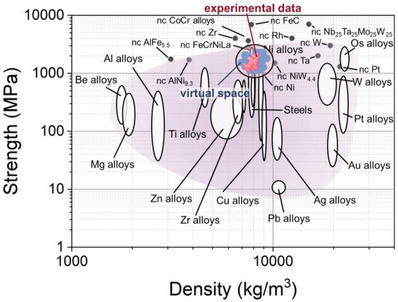
Comparison of the strength of metallic glasses and amorphous/crystalline composites from the CuAgZr system with other metals. Strength‐density Ashby chart for metals and alloys adapted from our previous work ref. [7] nc – nanocrystalline. (For interpretation of the references to color in this figure legend, the reader is referred to the web version of this article).

The application of combinatorial synthesis of MatLibs using specifically direct current magnetron sputtering (DCMS) and high‐throughput characterization methods has significant implications for determining the strength of new metallic glasses. By leveraging the vast amount of data generated by combinatorial synthesis and high‐throughput characterization, machine learning algorithms can identify patterns and relationships that may not be immediately apparent. This can significantly accelerate the discovery of new compositions that produce high‐strength metallic glasses. **Figure** **8** summarizes the achievements of this work and shows a strength‐density Ashby chart for metals and alloys. It presents alloys from the CuAgZr system produced experimentally and those for which predictions of properties in virtual space were made using a machine‐learning model. The density of the alloys was determined theoretically based on the content of Cu, Ag and Zr. Simultaneously, it should be noted that the actual density of the tested alloys may be lower due to the presence of oxygen and defects (e.g., inter‐columnar regions with looser packing). Conversion of hardness to strength was performed using the classical relationship H≈3*σ_y_*.^[^ ^23^ ^]^ However, it should be emphasized that this assumption is optimistic, at least for some alloys from the CuAgZr system, because the results of experimental studies sometimes showed a less favorable *H/σ_y_* ratio as high as 4.3 (e.g., Cu_45_Ag_14_Zr_51.3_ alloy, Figure 4). The metallic glasses and amorphous/crystalline composites of the CuAgZr system tested in this work have high strength, comparable to the best steels and nickel alloys. Moreover, the identification of alloys with similar properties can enable the selection of those that are more economically attractive, which can be of great importance in the development of new materials for commercial applications. Nevertheless, it should be stressed that the properties of alloys produced by PVD technology, can change significantly depending on the deposition parameters used.^[^ ^18^ ^]^ Indeed, in our recent work comparing the effects of DCMS and high‐power pulse magnetron sputtering (HiPIMS) on the structure and properties of alloys from the same system as the one studied in this work, i.e., CuAgZr, it was reported that for some compositions HIPIMS enabled up to 44% higher hardness.^[^ ^8^ ^]^

Of course, there arises a temptation to upscale the insights from thin films to the macro scale. Indeed, in our recent work,^[^ ^50^ ^]^ we compared the properties of a thin film metallic glass from the CuAgZr system, produced using the PVD method, with a bulk metallic glass created through arc melting with a similar chemical composition. Experiments were conducted at both room temperature and elevated temperatures up to 500 °C. The thin film metallic glass displayed enhanced thermal stability and a higher yield strength in every tested condition. These differences were attributed to a higher oxygen content present in the thin film metallic glass. To assess the utility of thin film MatLib in identifying high‐strength chemical compositions that can be upscaled to bulk form, it will be necessary to compare a larger set of thin film metallic glasses with their bulk counterparts, ensuring a similar contamination content.

## 4 Conclusions

This work focused on the investigation of mechanical properties of metallic glasses and amorphous/crystalline composites from the CuAgZr system. Combinatorial synthesis using DCMS was used to produce CuAgZr MatLib, and high‐throughput characterization methods were used to determine their mechanical properties and composition.

It was found that the high oxygen content in the Cu‐rich regions of the investigated CuAgZr MatLib is the result of post‐deposition oxidation of the inter‐columnar regions, with looser packing, in which oxygen can diffuse in a facilitated manner. This suggests that an important factor determining the oxygen content in synthesized alloys is the growth mechanism resulting from the mobility of atoms in a given system, which in turn is a function of chemical composition and process conditions.

The “Scanning Indenter” device, introduced in this study, significantly advances the state of the art and technological capabilities by enabling the automatic mapping of full wafers, facilitating the quantification of mechanical properties in MatLibs. It fosters the integration of various structural and mechanical characterization techniques such as XRF, XRD, and nanoindentation, promoting the creation of multimodal datasets for a more comprehensive understanding of materials properties and their potential applications.

Micropillar compression tests showed a profound influence of nanoscale structural features on plastic yielding and flow in the investigated alloys. An increase in atomic size mismatch is correlated with an increase in oxygen content and including oxygen in the calculations significantly increases atomic size mismatch. A linear relationship exists between hardness and atomic size mismatch for *δ_r_* ×100 < ≈20, indicating that hardness increases with increasing *δ_r_*. However, for alloys with a high oxygen content, observed for *δ_r_* ×100 > ≈20, there is a significant scatter in the hardness values, suggesting that the strongest alloys are those that can absorb large amounts of oxygen without having loosely packed regions, such as inter‐columnar regions, which can cause them to weaken.

A large number of ML algorithms have been tested and MLP has been shown to produce very satisfactory predictions for both training and testing sets. The leveraging of the fine‐tuned MLP algorithm enabled the prediction of the hardness of untested alloys in the virtual space of the investigated CuAgZr system, which can serve as a valuable guide for further exploration of this and other similar systems.

The results herein reported demonstrate that the combination of combinatorial synthesis, high‐throughput characterization, and machine learning methods holds significant promise for the discovery of new metallic glasses with improved strength and economic viability.

## 5 Experimental Section

### Material Synthesis

Thin film ternary CuAgZr compositionaly‐focused MatLib was fabricated in a vacuum chamber (Korvus Technology, UK) using PVD technique, specifically DCMS, onto a 4‐inch (100)‐oriented silicon substrate, coated with 100 nm thick amorphous silicon nitride. Co‐sputter deposition from high purity Cu, Ag, and Zr targets (HMW Hauner GmbH, 99.99%) led to a composition gradient across the wafer's surface. Sputtering was carried out in a chamber equipped with a rotary‐turbo pump combination to obtain a high base vacuum in the order of 6 × 10^−7^ mbar. Co‐sputtering was performed using Ar (purity of 99.9999%), at a working pressure of 5 × 10^−3^ mbar. The CuAgZr compositionally‐focused MatLib was synthesized in the form of 61 circular, 5 mm in diameter, patches using a 2 mm thick stainless‐steel mask. The substrate‐to‐target distance was optimized to 90 mm to achieve a desired composition gradient across the wafer. The deposition rate was ≈12 nm min^−1^. This compositionally‐focused MatLib was utilized for training the ML model. In addition, to validate the ML predictions in the virtual space, a second MatLib was fabricated, consisting of 21 patches. For the second MatLib, the deposition parameters were adjusted and calibrated to generate as wide a chemical composition gradient as possible. This broader gradient allowed for a more comprehensive exploration of the alloy system and provided a robust set of data for validating the predictive capabilities of the ML model.

### Chemical Composition and Thickness

The chemical composition and thickness of the deposited thin film was determined using the XRF spectrometry (Fischerscope X‐Ray XDV‐SDD by Fischer, Sindelfingen, Germany). A beam energy of 50 kV with a spot size of 0.3 mm was selected for the analysis (results are summarized in Table S2, Supporting Information). Due to the fact that the XRF method does not allow the determination of the oxygen content, additional experiments were carried out using the EDS to semiquantitatively determine the distribution of the oxygen in the analyzed MatLib. EDS experiments with an accelerating voltage of 30 kV were performed in the Mira‐3 scanning electron microscope (SEM) (Tescan, Brno, the Czech Republic), equipped with the Octane Plus SDD detector (EDAX, Mahwah, USA). Chemical analysis was carried out using a ZAF correction (results are summarized in Table S2, Supporting Information). The EDS method was characterized by low accuracy in oxygen quantification. It should also be noted that the uncertainty of oxygen measurement was much greater in the case of alloys with a low oxygen content. A direct comparison of the oxygen content between different MatLib regions was additionally made difficult by the fact that the volume of interaction between the electron beam and the sample depends on the chemical composition. Hence, the chemical information in different areas of MatLib was obtained from different interaction volumes. Nevertheless, global semiquantitative trends in the change in oxygen content can be captured. The EDS results were summarized in Table S1 (Supporting Information). To validate these trends, quantitative oxygen analysis was performed using ERDA and RBS on six selected samples at the 1.7 MV Tandetron accelerator facility of the Laboratory of Ion Beam Physics at ETH Zurich. For the ERDA analysis a 13 MeV ^127^I beam was used under a scattering angle ϕ of 36° and the scattered recoils were identified by the combination of a time‐of‐flight spectrometer with a gas ionization chamber.^[^ ^51^ ^]^ Precise measurement of Ag content by ERDA was difficult due to the background generated by the primary iodine beam, therefore, only Cu, Zr, O and other light elements (if detected) depth profiles had been extracted from heavy ion ERDA measurements using the POTKU software.^[^ ^52^ ^]^ A decrease of heavy element concentrations (Zr in particular) with sample depth was observed, which was an artifact due to multiple scattering of the recoil ion.

Since RBS gives more reliable results for heavy elements, additional RBS experiments were performed to measure the Ag content and confirm the ERDA results for the other metallic constituents. Consequently, a more accurate estimation of the oxygen content in the tested samples was achieved. The RBS measurements were obtained using a 2 and 5 MeV He beam under a backscattering angle θ of 167.5° and the data were analyzed using the RUMP software^[^ ^53^ ^]^ considering Cu, Ag and Zr. The RBS beam spot had an area of ≈1 mm^2^. The circular samples were hit at the center ± 1 mm. The RBS/ERDA chemical composition, listed in Table S2 (Supporting Information), was normalized to RBS results via the Cu content. Values for O, Cu, Zr, and Ag add up to 100%. The oxygen‐rich top layer (the first few tens of nm) was excluded from the quantification. H, C, and N which have also been found in some samples are not included. All the RBS/ERDA experiments were conducted under vacuum conditions ranging for 10^−7^ – 10^−6^ mbar.

Additionally, the distribution of elements in the microstructure of selected alloys was studied by Themis 200 G3 spherical aberration (probe) corrected Transmission Electron Microscope (TEM) (Thermo Fischer, Waltham, USA) operating at 200 kV. The TEM studies were performed on TEM lamellae, prepared via focused ion‐milling (FIB technique) using a dual beam FIB‐SEM Tescan Lyra FEG system (Brno, the Czech Republic). The concentration line profiles, and the elemental maps were acquired using EDS in STEM mode.

### Phase Analysis

To determine the structure of investigated alloys, 10 selected patches were measured using XRD. The measurements were carried out with CuK_a1_ and CuK_a2_ radiations (λ = 1.5406 and 1.54439 Å, respectively) by means of the D8 Discover diffractometer (Bruker, Billerica, USA), equipped with programmable sample‐positioning stage. The phase composition of the CuAgZr MatLibs was identified using XRD data in the 2θ range from 30 to 90° obtained under the conditions: voltage of 40 kV, current of 40 mA, step size of 0.02° and the collection time at each step of 2 s. The θ/2θ‐scans were performed with an offset of −4° from the symmetrical diffraction geometry to avoid a too high intensity from the (400) reflection of the oriented (100) single crystal Si substrate.

### Nanoindentation Mapping

To extract the mechanical properties of all 61 patches (see Section 2.1), and to provide a statistically sound datasets, a “Scanning Indenter” – setup, built around the components of the portable micro‐ and nanoindenter from Alemnis (Thun, Switzerland), was developed. It allows the automated mapping of a full 4‐inch wafer, and, thus, the quantification of mechanical properties in the compositionally‐focused MatLibs. The system can accommodate the full wafer without the need of cutting the sample into dedicated pieces and gluing them onto SEM stubs for the experiments (Figure S4, Supporting Information), as necessary for other commonly used systems. This serves as a unique trademark of the system. It thus, saves a significant amount of time, and also allows subsequent mapping experiments capturing different conditions, e.g., when quantifying the effect of heat treatments or different deposition conditions. Hence, it provides the opportunity to characterize a large number of different conditions in a single process, opening up new high‐throughput possibilities in the search of novel material systems. Furthermore, it facilitates combining structural and mechanical characterization techniques (XRF, XRD, nanoindentation) in creating multimodal datasets. More details about the “Scanning Indenter” can be found in Supporting Information.

For the actual experiments, 9 Berkovich‐indents were performed in a 3×3 array in the center of each of the 61 patches (total 549 indents, Figure S5, Supporting Information), with an indent‐spacing of 20 µm. Indents were done with a displacement‐controlled protocol at a displacement rate of 10 nm^−1^ s and up to a maximum indentation depth of 220 nm (< 10% of the CuAgZr layer thickness). A mapping protocol was defined based on the design of the prepared sample (Figures S1 and S2, Supporting Information). To ensure that any potential sample tilt does not interfere with the measurements, i.e., primarily to avoid scratching the surface with the tip while travelling between indentation locations, an x‐y tilt plane was determined before the actual mapping started. Based on the corresponding angles in x and y, the tip moved either up or down in‐between indents (safety travel). During the test, load and displacement data were recorded. Data were analyzed with custom‐written codes in Python and R as well as the commercially available software AMMDA from Alemnis (Thun, Switzerland). Post‐processing steps included data structuring, calculation of hardness and elastic modulus^[^ ^54^ ^]^ calculation of mean, standard deviation. The instrument compliance and tip area function were determined based on continuous stiffness measurements on a calibration wafer of fused silica. Coefficients to estimate the tip shape were done according to the Oliver‐Pharr method.^[^ ^54^ ^]^ An overview of plots with all 3×3 arrays of indents on each of the 61 patches can be found in the Figure S5 (Supporting Information). The mean values along with the standard deviation of the nanoindentation results are summarized in Table S1 (Supporting Information).

### Micropillars Compression Tests

To measure the strength and to determine the *H/σ_y_* ratio of the MG thin‐film micropillars, compression tests were performed on selected alloys. Determination of *H/σ_y_* ratios was important considering a conversion of hardness into the strength of CuAgZr alloys. Micropillars with diameters of 1.33 ± 0.17 µm and height 2.98 ± 0.14 µm were fabricated from the selected regions of the CuAgZr MatLib using a dual beam FIB‐SEM Tescan Lyra FEG system. The dimensions of the micropillars were selected to obtain small aspect ratio (≈2.2) and avoid their buckling during micropillars compression tests.^[^ ^55^ ^]^ The taper angle of the micropillars was 3.39 ± 0.38°. Both dimensions and taper were measured from SEM images. To estimate the strength of each of the investigated alloys at least three micropillars were compressed. The micropillars compression tests were performed using an in situ nanoindenter Alemnis, equipped with a 5 µm flat punch diamond tip, and installed in the SEM (Philips XL30 ESEM FEG, Amsterdam, Netherlands). The micropillars were compressed under the displacement‐controlled mode at strain rate of 10^−3^ s^−1^. Before converting the load‐displacement curves into engineering stress‐strain curves correction for instrument and compliances were calculated.^[^ ^56^ ^]^ The stress value at 0.2% strain offsets of the engineering stress–strain curves was taken as the yield strength.

### Forecasting the Strength of Untested Alloys in Virtual Space

**Figure 9:**
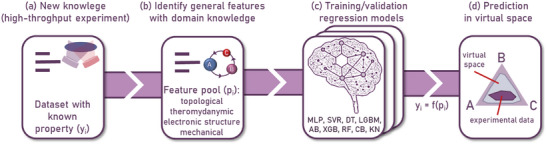
A schematic illustrating the process of creating ML models used in this work. (a) In the first step, a high‐throughput experiment was used to create a database in which the target property is hardness (y_i_). (b) General features were created based on chemical composition and domain knowledge. (c) Testing of the regression models along with the coarse refinement of hyperparameters. (d) Finally, the optimized model was used to predict the hardness of unexplored alloys in virtual space.

To predict the strength of unexplored Cu‐Ag‐Zr alloys in virtual space the best‐performing ML model was employed. The performance of each model was evaluated using the root mean squared error (RMSE), which was defined as:

(3) $$RMSE=\sqrt{\frac{1}{n}\sum_{i=1}^{n}{\left(y_{i}-\hat{y_{i}}\right)}^{2}}$$

where *y_i_* is the true value and $\hat{y_{i}}$ is the predicted value. A schematic illustrating the process of creating machine learning models used in this work is shown in **Figure** **9**. The dataset used in the study was obtained from a high‐throughput characterization of the CuAgZr MatLib and consisted of 61 samples in which the target property (*y_i_*) was hardness (Figure 9a). The set of features was created based on domain knowledge using topological, thermodynamic, electronic structure, and mechanical attributes, which correlate with the structure and properties of metallic materials^[^ ^36^, ^57^, ^58^, ^59^, ^60^, ^61^, ^62^, ^63^, ^64^, ^65^, ^66^, ^67^, ^68^, ^69^ ^]^ (Figure 9b and **Table** **1**). The formulas for these features are summarized in Table S6 (Supporting Information). To engineer the features and evaluate the performance of the regression models for prediction of the material properties the open‐source scikit‐learn library was used.^[^ ^70^ ^]^ The Pearson correlation analysis was conducted to detect dependencies among the features built based on domain knowledge that was helpful in reducing the dimensionality of the data by removing redundant and/or highly correlated features. The final set of features was then standardized, and the data were split into training and testing sets. During standardization, the data was transformed so that its distribution had a mean of 0 and a standard deviation of 1. This helped equalize the range and distribution of features and ensure that each feature contributed equally to the distance metric used in the machine learning algorithms. In a material design problem where the available data was limited, as is the case in this study with a dataset of only 61 samples, the primary focus was on capturing the overall trend for untested alloy compositions rather than solely minimizing the disparity between the predicted and experimental values. The goal was to achieve generalizability in the predictions made by the model. However, in order to find the most effective model, a large number of regressors were tested (Figure 9c). The algorithms used in the study included K‐Nearest Neighbors Regression (KN),^[^ ^27^ ^]^ Support Vector Regression (SVR),^[^ ^28^ ^]^ Multilayer Perceptron Regression (MLP),^[^ ^29^ ^]^ XGBoost Regression (XGB),^[^ ^30^ ^]^ Decision Tree Regression (DT),^[^ ^31^ ^]^ Random Forest Regression (RF),^[^ ^32^ ^]^ AdaBoost Regression (AB),^[^ ^33^ ^]^ LightGBM Regression (LGBM),^[^ ^34^ ^]^ and CatBoost Regression (CB).^[^ ^35^ ^]^ In ML, an excess of features compared to training data can lead to a model that overfits, negatively impacting its performance on unseen data. To address this challenge, a thorough approach to hyperparameter optimization was strategically employed. The strategy was focused on avoiding a scenario in which the model could memorize the training data without truly understanding the underlying patterns. In this context, it was carried out multiple iterations of the grid search. In each successive iteration, the hyperparameters were thoughtfully adjusted with the aim of reducing the RMSE. The details of tested hyperparameters from the ultimate iteration can be found in Table S7 (Supporting Information). To further enhance the reliability of the model and mitigate the effects of random fluctuations, each model configuration was ran ten times with different random states, averaging the outcomes. This comprehensive process involved testing more than 100 000 different configurations, ensuring a wide exploration of the hyperparameter space. Ultimately, the most effective algorithm, the MLP, was singled out. This algorithm was then fine‐tuned even further and used for predicting the hardness of untested alloys within a virtual space, as visualized in Figure 9d. However, it was important to recognize that there might be a combination of hyperparameters that could potentially offer better performance for other models. Despite this, based on extensive explorations and optimization, the MLP algorithm demonstrated the most exceptional performance. The potential of other models to possibly outperform the MLP with a different hyperparameter setup was acknowledged. Still, within the vast hyperparameter space that was investigated, none of the other configurations proved superior to the MLP. This observation does not depreciate the potential of these other models but rather underscores the effectiveness of the selected model within the tested hyperparameter confines. Future studies might wish to delve further into this space or incorporate other novel models for comparative evaluation against the MLP.

**Table 1:** A feature pool built on domain knowledge used in machine learning regression models. Note that S_E_/k_B_ and Λ can also be considered as thermodynamic parameters.

Class	Feature pool
Topological	**δ_r_** – atomic size mismatch,^[^ ^36^ ^]^ **D.r** – local atomic size mismatch,^[^ ^57^ ^]^ **S_E_/k_B_** – excess configurational entropy,^[^ ^62^, ^63^ ^]^ **Λ –** entropy and atomic size ratio,^[^ ^9^, ^64^ ^]^ **γ –** the largest and the smallest atom,^[^ ^9^, ^65^ ^]^
Thermodynamic	**ΔH_mix_ –** enthalpy of mixing in liquid phase,^[^ ^63^ ^]^ **Ω** – competition between entropy and enthalpy of mixing in liquid phase,^[^ ^9^, ^66^ ^]^ **ϕ** – entropy effect gauge,^[^ ^9^, ^67^ ^]^ **Φ_f_** – competition between entropy and enthalpy of formation of binary compounds,^[^ ^9^, ^68^ ^]^
Electronic structure	**VEC_LM_ –** local mismatch of valence electron concentration,^[^ ^9^ ^]^ **e/a_LM_** – local mismatch of itinerant electron concentration,^[^ ^9^ ^]^ **χAllen_LM_** – local mismatch of Allen's electronegativity^[^ ^69^ ^]^
Mechanical	**η** – modulus mismatch in strengthening model,^[^ ^58^ ^]^ **A –** energy term in strengthening model,^[^ ^59^ ^]^ **F –** Peierls‐Nabarro factor,^[^ ^60^ ^]^ **w** – six square of work function,^[^ ^61^ ^]^ **G** – shear modulus,^[^ ^60^ ^]^ **δG** – shear modulus mismatch,^[^ ^60^ ^]^ **D.G** – local size shear modulus mismatch,^[^ ^60^ ^]^ **µ** – lattice distortion energy^[^ ^60^, ^71^ ^]^

## Conflict of Interest

The authors declare no conflict of interest.

## Author Contributions

K.W. conceived the project and designed the research, performed XRF, XRD, and EDS experiments, developed machine learning model to predict Cu‐Ag‐Zr alloys strength, analyzed and interpreted all data, wrote the original draft; J.S. and A.G. developed “Scanning Indenter”; A.G. and F.F.K performed nanoindentation experiment; KW. and K.P. performed micropillars compression tests with help from M.J.; K.P. contributed to the selection of alloys with high GFA; A.M.M. and C.V. performed ERDA and RBS experiments; A.S. synthesized CuAgZr material library and performed TEM experiments; supervision was performed by J.M.; all authors discussed the results and commented on the manuscript.

## Supporting information

Supporting Information

## Data Availability

The data that support the findings of this study are available in the supplementary material of this article.
