# Supplementary material for: Unlocking the Potential of CuAgZr Metallic Glasses: A Comprehensive Exploration with Combinatorial Synthesis, High‐Throughput Characterization, and Machine Learning
Source: Adv Sci (Weinh). 2023 Sep 23;10(31):2302997. doi: 10.1002/advs.202302997 (PMC10625089; doi:10.1002/advs.202302997)
Supplement: Supplementary file 1 — Supporting Information [file ADVS-10-2302997-s001.pdf]

ADVANCED SCIENCE
Open Access

Supporting Information

for *Adv. Sci.*, DOI 10.1002/advs.202302997

Unlocking the Potential of CuAgZr Metallic Glasses: A Comprehensive Exploration with Combinatorial Synthesis, High-Throughput Characterization, and Machine Learning

Krzysztof Wieczerzak, Alexander Groetsch, Krzysztof Pajor, Manish Jain, Arnold M. Müller, Christof Vockenhuber, Jakob Schwiedrzik, Amit Sharma, Fedor F. Klimashin and Johann Michler*

Supporting Information

Unlocking the potential of CuAgZr metallic glasses: A comprehensive exploration with combinatorial synthesis, high-throughput characterization, and machine learning

Krzysztof Wieczerzak, Alexander Groetsch, Krzysztof Pajor, Manish Jain, Arnold M. Müller, Christof Vockenhuber, Jakob Schwiedrzik, Amit Sharma, Fedor F. Klimashin, and Johann Michler*

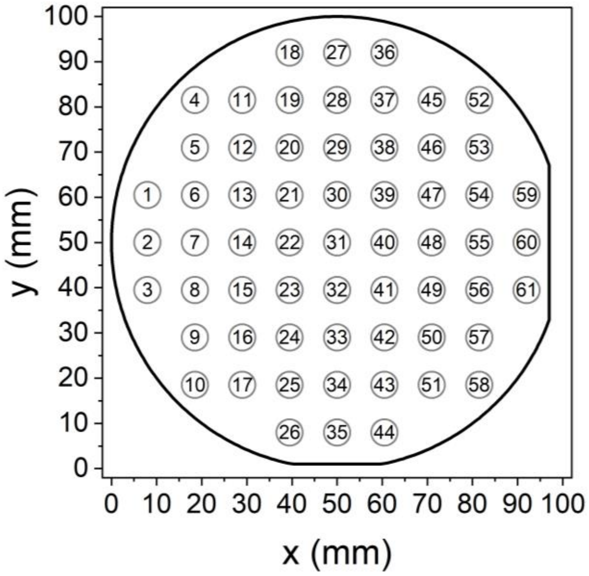

Figure S1. Marking of patches on the tested material library.

Table S1. Chemical composition, measured with XRF and EDS, and nanoindentation results (mean ± standard deviation) measured at the center of each of the 61 patches of the investigated CuAgZr material library.

No	XRF				EDS				Nanoindentation	
	Thickness (µm)	Cu (at. %)	Ag (at. %)	Zr (at. %)	O (at. %)	Cu (at. %)	Ag (at. %)	Zr (at. %)	H (GPa)	Er (GPa)
**1**	5.05	25.4	4.3	70.3	3.5	27.2	2.2	67.1	4.1 ± 0.1	91.2 ± 3.5
**2**	5.08	27.8	4.0	68.2	2.9	30.1	1.9	65.1	4.0 ± 0.1	91.4 ± 7.4
**3**	5.23	31.1	3.9	65	4.7	32.3	1.8	61.2	4.0 ± 0.2	90.3 ± 4.3
**4**	4.69	25.0	6.2	68.8	4.7	26.7	3.7	64.9	3.8 ± 0.2	87.9 ± 4.4
**5**	4.77	27.4	4.9	67.7	3.7	29.0	2.9	64.4	3.9 ± 0.2	92.7 ± 3.9
**6**	4.84	29.8	4.5	65.7	4.0	31.2	2.5	62.3	3.9 ± 0.2	88.1 ± 6.3
**7**	4.89	32.7	4.5	62.8	6.2	33.6	2.3	57.9	3.8 ± 0.2	92.7 ± 3.8
**8**	4.91	35.9	4.1	60	7.7	35.9	2.1	54.3	3.9 ± 0.2	94.1 ± 9.2
**9**	4.91	38.7	4.3	57	10.8	37.7	2.1	49.4	5.3 ± 0.3	105.1 ± 5.2
**10**	4.61	44.1	3.9	52	11.0	42.6	2.0	44.4	4.3 ± 0.4	104.5 ± 8.5
**11**	4.35	28.7	7.2	64.1	3.8	30.7	4.8	60.7	3.5 ± 0.1	94.6 ± 3.1
**12**	4.44	31.0	6.2	62.8	4.0	32.6	3.9	59.5	3.8 ± 0.1	99.7 ± 2.9
**13**	4.54	33.9	5.4	60.7	4.6	35.7	3.1	56.6	3.9 ± 0.2	102.5 ± 4.2
**14**	4.65	37.1	4.8	58.1	7.7	37.4	3.0	51.9	4.0 ± 0.3	99.1 ± 3.9
**15**	4.68	40.7	4.7	54.6	11.8	38.7	2.6	46.9	5.3 ± 0.3	118.6 ± 3.0
**16**	4.74	44.5	4.7	50.8	13.1	41.6	2.4	42.9	4.8 ± 0.3	120.3 ± 5.2
**17**	4.72	48.1	4.2	47.7	12.8	44.8	2.1	40.3	4.4 ± 0.5	107 ± 10.4
**18**	3.99	29.7	9.1	61.2	5.0	31.2	7.0	56.8	3.3 ± 0.2	96.4 ± 3.1
**19**	4.05	32.2	8.4	59.4	4.0	34.2	5.9	55.9	3.5 ± 0.2	101.0 ± 5.1
**20**	4.20	35.4	7.1	57.5	3.9	37.3	5.1	53.7	3.8 ± 0.1	103.4 ± 2.9
**21**	4.25	38.8	6.0	55.2	5.6	39.6	4.0	50.8	3.7 ± 0.2	102.9 ± 5.5
**22**	4.44	41.4	6.0	52.6	9.8	41.1	3.3	45.8	4.4 ± 0.1	106.1 ± 1.8
**23**	4.50	45.6	5.3	49.1	13.3	42.7	2.9	41.1	5.1 ± 0.2	121.3 ± 3.4
**24**	4.70	49.3	4.8	45.9	14.2	45.5	2.5	37.8	4.6 ± 0.4	117.3 ± 6.9
**25**	4.73	53.3	4.3	42.4	14.7	48.6	2.1	34.6	4.3 ± 0.3	102.9 ± 4.2
**26**	4.66	56.6	4.1	39.3	14.7	50.9	1.9	32.5	4.1 ± 0.4	96.7 ± 7.9
**27**	3.81	32.7	10.9	56.4	4.1	35.0	8.5	52.4	3.8 ± 0.2	100.4 ± 4.0
**28**	4.04	35.5	9.8	54.7	3.8	37.9	7.5	50.8	3.7 ± 0.2	107.9 ± 4.9
**29**	4.01	39.1	8.3	52.6	4.3	41.0	6.2	48.5	3.8 ± 0.1	108.5 ± 4.1
**30**	4.13	42.5	6.8	50.7	5.9	43.7	4.8	45.6	3.9 ± 0.2	109.5 ± 4.1
**31**	4.31	46.4	6.2	47.4	12.8	43.7	3.8	39.7	5.1 ± 0.2	118.2 ± 4.4
**32**	4.43	50.6	5.6	43.8	15.7	45.4	3.2	35.7	4.9 ± 0.4	115.4 ± 5.8
**33**	4.54	54.2	5.2	40.6	15.6	48.9	2.7	32.8	4.2 ± 0.3	102.8 ± 4.2
**34**	4.60	58.2	4.5	37.3	17.0	51.5	2.2	29.3	3.9 ± 0.4	90.9 ± 8.4
**35**	4.58	60.8	4.3	34.9	17.4	53.7	1.8	27.1	3.6 ± 0.6	91.7 ± 8.1
**36**	3.55	35.7	13.0	51.3	4.0	37.6	11.2	47.2	4.1 ± 0.1	110.6 ± 4.5
**37**	3.63	38.7	11.4	49.9	4.4	41.0	9.2	45.4	4.1 ± 0.1	115.8 ± 5.9
**38**	3.75	42.4	9.4	48.2	6.0	44.2	6.7	43.1	4.2 ± 0.2	113.0 ± 4.0

**39**	3.80	47.0	7.6	45.4	8.8	46.4	5.1	39.7	4.3 ± 0.2	111.9 ± 5.4
**40**	3.98	50.6	6.9	42.5	17.1	45.7	3.9	33.3	5.9 ± 0.3	127.7 ± 3.9
**41**	4.11	55.1	5.9	39	18.2	48.0	3.3	30.5	4.8 ± 0.2	114.1 ± 5.5
**42**	4.20	58.5	5.0	36.5	18.2	50.9	3.0	27.9	3.8 ± 0.4	94.9 ± 7.8
**43**	4.37	62.3	4.8	32.9	18.4	53.9	2.2	25.5	3.8 ± 0.2	94.6 ± 2.8
**44**	4.44	65.7	3.9	30.4	17.8	56.9	1.9	23.4	3.7 ± 0.2	92.8 ± 4.5
**45**	3.45	41.9	12.7	45.4	7.6	42.7	10.3	39.4	4.4 ± 0.2	115.0 ± 5.1
**46**	3.55	46.3	10.6	43.1	7.6	46.6	8.2	37.6	4.3 ± 0.1	117.3 ± 3.8
**47**	3.68	50.7	8.4	40.9	10.6	48.9	6.1	34.4	4.5 ± 0.1	114.9 ± 6.2
**48**	3.85	54.4	7.5	38.1	17.4	48.8	4.7	29.1	5.6 ± 0.5	126.2 ± 3.5
**49**	4.00	58.7	6.2	35.1	17.2	52.1	4.0	26.7	4.2 ± 0.2	108.7 ± 5.7
**50**	4.13	62.8	5.3	31.9	17.2	54.9	3.2	24.7	4.2 ± 0.4	100.8 ± 3.1
**51**	4.31	66.7	4.6	28.7	17.6	57.8	2.6	22	4.4 ± 0.3	107.7 ± 7.6
**52**	3.34	45.0	14.0	41	4.3	46.5	12.0	37.2	4.9 ± 0.2	115.9 ± 6.8
**53**	3.44	49.3	11.8	38.9	6.9	50.1	9.4	33.6	4.8 ± 0.2	120.3 ± 5.4
**54**	3.57	54.0	9.5	36.5	12.2	51.2	6.9	29.7	4.5 ± 0.2	121.9 ± 4.7
**55**	3.72	58.0	8.1	33.9	17.1	51.4	5.2	26.3	5.8 ± 0.3	122.5 ± 4.3
**56**	4.15	62.7	6.8	30.5	17.1	54.8	4.2	23.9	5.0 ± 0.5	114.3 ± 7.3
**57**	4.36	66.3	5.4	28.3	16.6	58.2	3.3	21.9	5.2 ± 0.3	111.7 ± 4.7
**58**	4.55	69.7	4.6	25.7	16.7	61.0	2.6	19.7	5.5 ± 0.3	114.3 ± 4.1
**59**	3.51	56.6	10.4	33	10.6	54.4	7.8	27.2	4.9 ± 0.3	114.8 ± 4.5
**60**	3.43	62.6	6.6	30.8	16.6	54.6	5.6	23.2	6.2 ± 0.3	121.5 ± 4.4
**61**	3.87	65.3	7.1	27.6	19.0	56.0	4.2	20.8	6.3 ± 0.3	123.9 ± 6.9

Table S2. Chemical composition of selected regions of CuAgZr material library determined with RBS and ERDA and yield strength (σ_y), determined via in situ micropillars compression tests.

	**RBS**			**ERDA**				**σ_y (GPa)**
**No**	Cu (at. %)	Ag (at. %)	Zr (at. %)	Cu (at. %)	Ag (at. %)	Zr (at. %)	O (at. %)	
**1**	25.2	1.5	73.3	28.5	1.5	67.2	2.8	0.98 ± 0.119
**14**	37.8	2.0	60.2	38.0	2.0	56.4	3.6	1.30 ± 0.064
**36**	35.1	9.7	55.2	36.4	8.9	53.6	1.0	1.35 ± 0.077
**42**	59.5	2.1	38.4	47.5	2.1	29.4	21.1	0.89 ± 0.002
**52**	47.2	9.8	43.0	47.8	8.9	42.3	1.0	1.15 ± 0.153
**59**	60.2	6.1	33.7	54.0	5.8	31.0	9.2	1.74 ± 0.050

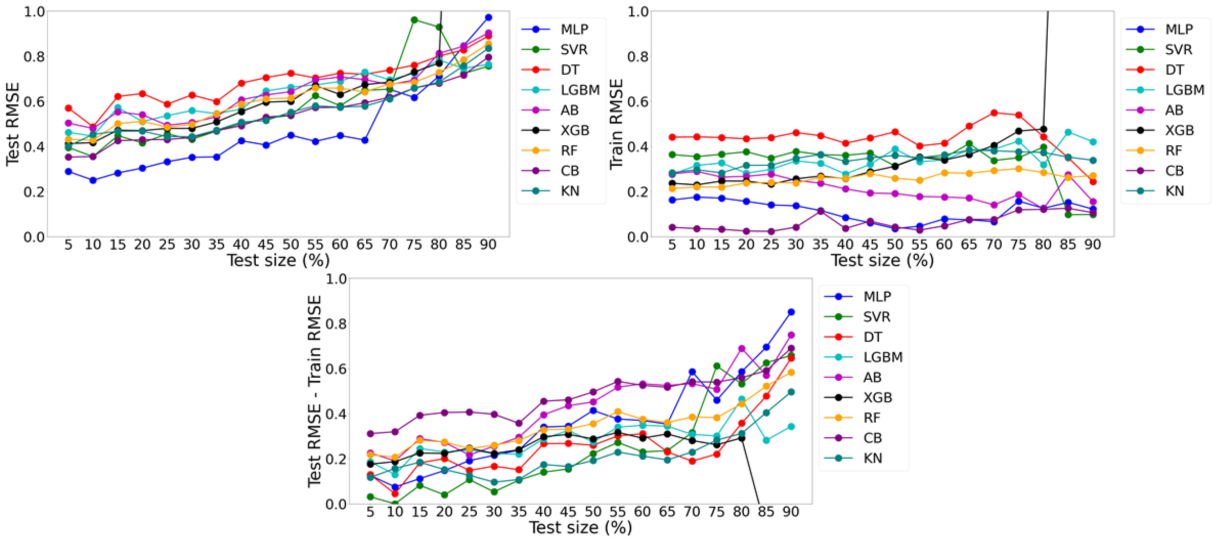

Figure S2. Test RMSE, train RMSE and overfitting/underfitting (test RMSE – train RMSE) for various machine learning models as a function of test set size.

Table S3. The best feature set utilized in an MLP model and the root mean squared error (RMSE) of the model's predictions.

**Number of features**	**The best feature set**	**RMSE**
**1**	μ	0.4524
**2**	Φ_f, μ	0.4270
**3**	Φ_f, η, μ	0.4002
**4**	Φ_f, χAllen_{LM}, D.G, μ	0.4108
**5**	Φ_f, χAllen_{LM}, η, D.G, μ	0.3851
**6**	δ, Φ_f, χAllen_{LM}, η, D.G, μ	0.3847
**7**	δ, D.r, φ, Φ_f, χAllen_{LM}, D.G, μ	0.3861
**8**	δ, D.r, Φ_f, χAllen_{LM}, η, δG, D.G, μ	0.3846
**9**	δ, D.r, SE/kB, Φ_f, χAllen_{LM}, η, δG, D.G, μ	0.3863
**10**	δ, D.r, SE/kB, φ, Φ_f, χAllen_{LM}, η, δG, D.G, μ	0.3891

Table S4. Chemical composition measured by XRF and nanoindentation results (mean ± standard deviation) measured in the middle of each of the 21 patches of the CuAgZr material library used for validating machine learning predictions of alloy properties, with chemical compositions located in the virtual space.

No	XRF				Nanoindentation
	Thickness (μm)	Cu (at. %)	Ag (at. %)	Zr (at. %)	H (GPa)
**1**	3.72	23.2	11	65.8	2.6 ± 0.1
**2**	4.22	16.1	12.1	71.8	2.8 ± 0.3
**3**	4.31	11.9	16.1	72	5.2 ± 0.2
**4**	3.05	50.5	10.9	38.6	3.2 ± 0.1
**5**	3.07	36.6	14.7	48.7	2.2 ± 0.2
**6**	3.12	25.4	20.2	54.4	2.6 ± 0.4
**7**	3.21	17.4	26.1	56.5	3.8 ± 0.2
**8**	3.17	12.2	35.9	51.9	3.7 ± 0.5
**9**	3.03	64.9	11.8	23.3	5.7 ± 0.2
**10**	2.73	50	18.6	31.4	5.5 ± 0.2
**11**	2.7	34.5	27.8	37.7	4.2 ± 2.5
**12**	2.78	22.3	40.4	37.3	5.4 ± 0.2
**13**	3.02	14.1	53.2	32.7	5 ± 0.2
**14**	3.2	75	11.3	13.7	4.7 ± 0.2
**15**	2.69	58.6	21.7	19.7	5.7 ± 0.2
**16**	2.56	40.2	37	22.8	5.4 ± 0.3
**17**	2.81	23.9	53.7	22.4	4.8 ± 0.2
**18**	3.4	13.7	68.6	17.7	4.6 ± 0.3
**19**	2.72	64.5	23.6	11.9	5.1 ± 0.1
**20**	2.63	43.1	42.7	14.2	3.6 ± 0.3
**21**	3.02	24.8	62.4	12.8	4.2 ± 0.3

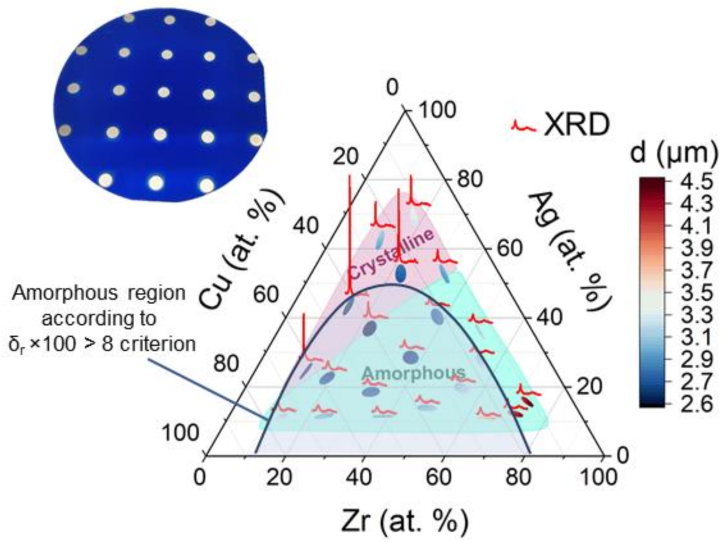

Figure S3. New material library of 21 patches produced to validate the effectiveness of the δ_r parameter in identifying the location of amorphous regions. Each of the ellipses in the figure illustrates the actual gradient in the patch under study and a corresponding diffractogram is included above each one.

The scanning indenter

The scanning indenter (Figure S4) is built around the components of the portable micro- and nanoindenter from Alemnis (Thun, Switzerland). The main components of the system include the tip (Synthon), a tip-side load cell (Alemnis), a displacement head with actuator for tip movement (SmarAct), a sample stage for x- and y-movement with picometer precision (SmarAct), a microscope with digital zoom (Thorlabs), a vacuum jug for sample mounting including a vacuum pump, a passively damped optical table (ThorLabs), a light-blocking encasing box, and a specifically designed software dedicated to the mapping of large arrays (Alemnis with validation work by Empa). A reference indent was performed before the actual mapping experiments to determine the offset between the Berkovich tip and the microscope objective (tip-optics calibration). The system was validated before the actual experiments, which included the repeatability of such tip-optics calibration measurements and to estimate the influence of the vacuum pump on the noise level of the load and displacement signals. Positioning accuracy was observed to be in the range of 1-2 μm. Tests for the noise level were performed on a Si wafer with one unpolished side to increase friction between the wafer and the sample holder for tests when the vacuum pump was off. Indents were done on the polished side of the Si wafer with load- and displacement-controlled protocols over a range of 4 mN to 20 mN and 155 nm to 455 nm, respectively. Nanoindentation curves are shown in Figure S5. Tests with the Berkovich tip in air (without contact) were performed as well (sampling rate: 50 kHz). No significant difference ($p=0.05$) was found for the root-mean square (RMS) and peak-to-peak noise for both the load and displacement signals (Table S5, (Figures S6 and S7)).

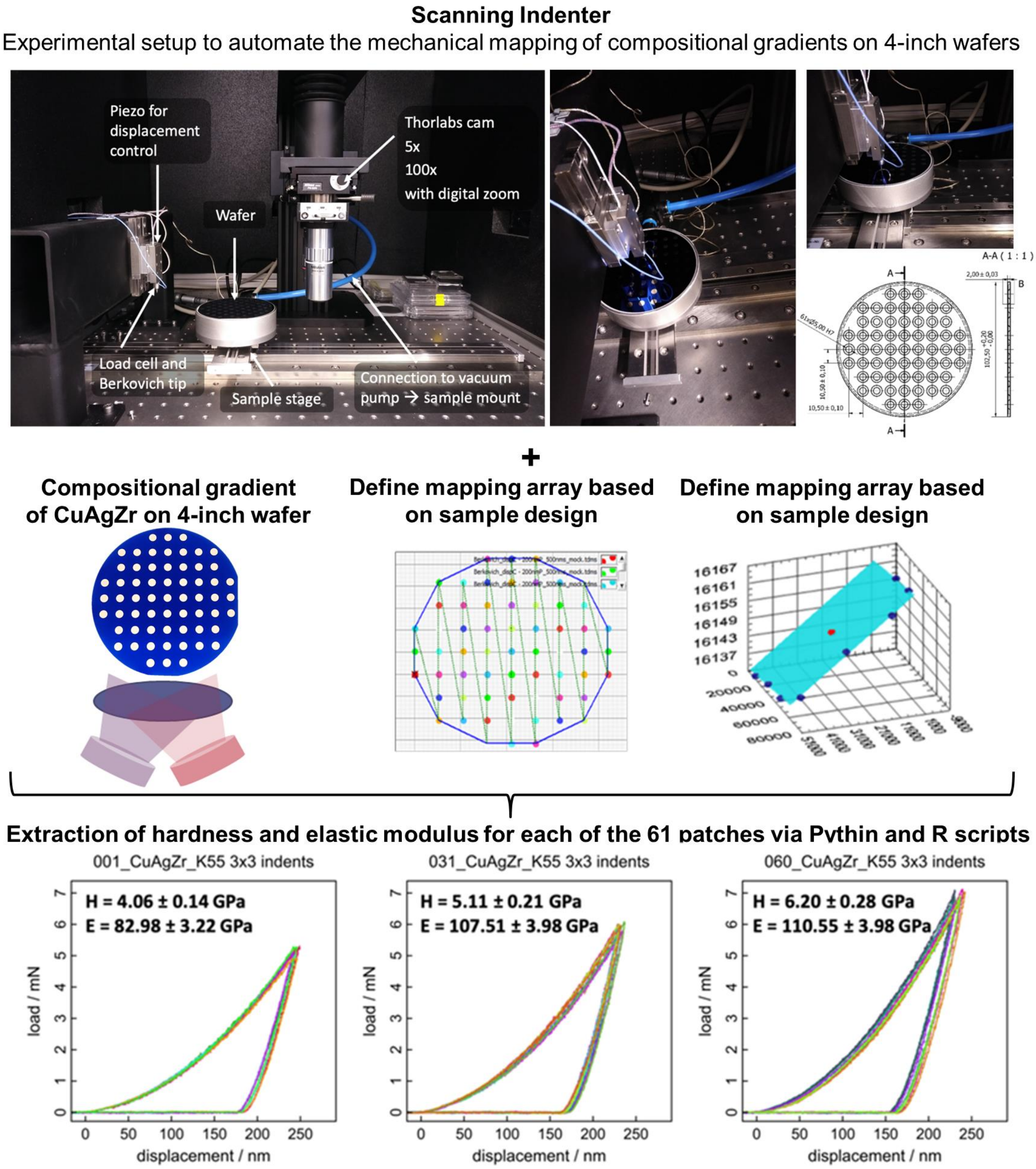

Figure S4. Scanning indenter for an automated mechanical mapping of compositional gradients on 4-inch wafers. Top: Experimental setup and major components that includes the nanoindenter with the displacement actuator, the tip and the load cell, the sample mount on an x- and y-stage and the optical microscope. Middle: Based on the sample design for the material synthesis, a mapping array with 61 patches was defined with a 3×3 indentation sub-array on each patch. Bottom: Overlay of load-displacement curves for three exemplary patches (1, 31, 61).

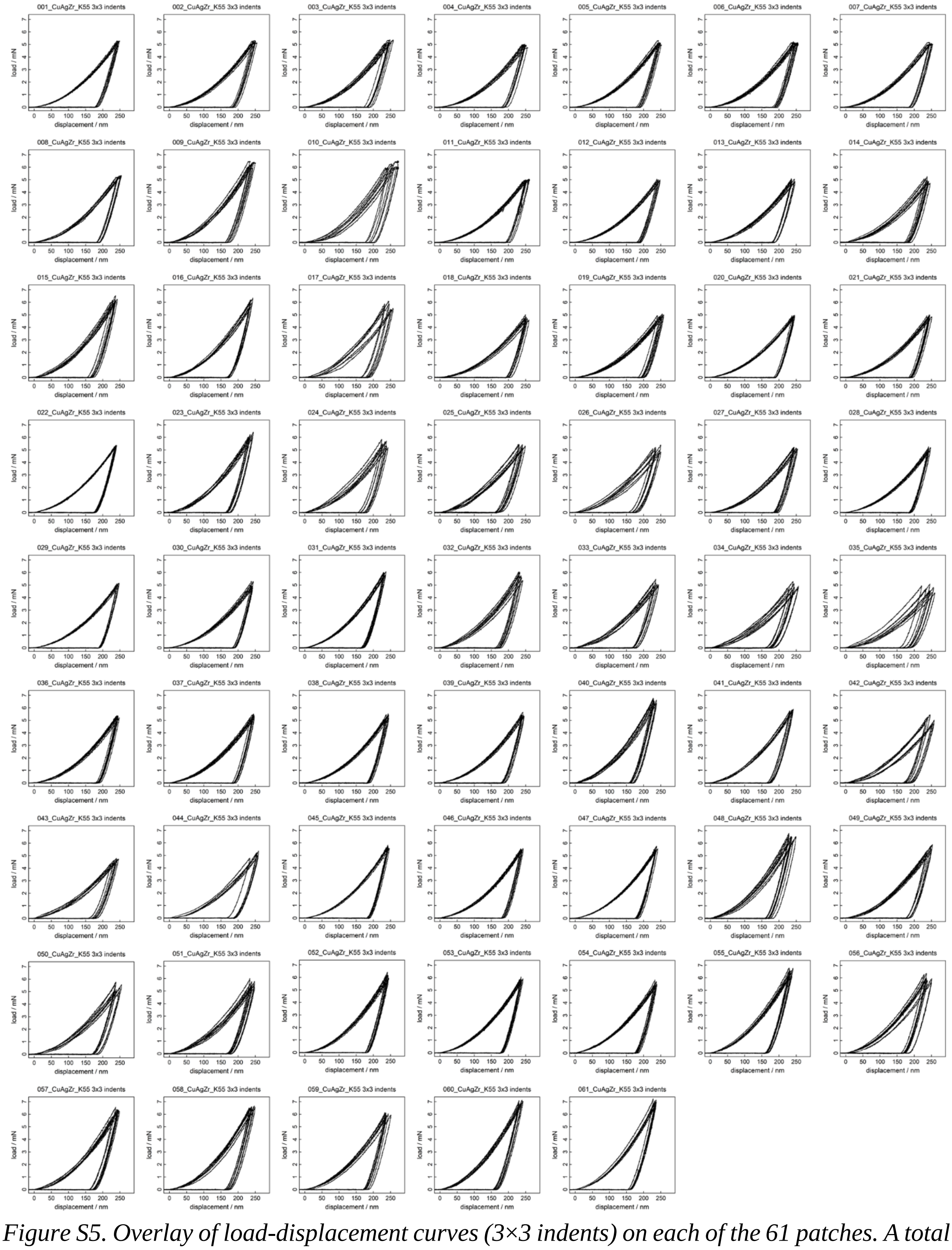

Figure S5. Overlay of load-displacement curves (3×3 indents) on each of the 61 patches. A total of 549 indents were performed.

Details on the noise measurements (influence of vacuum pump for sample mount)

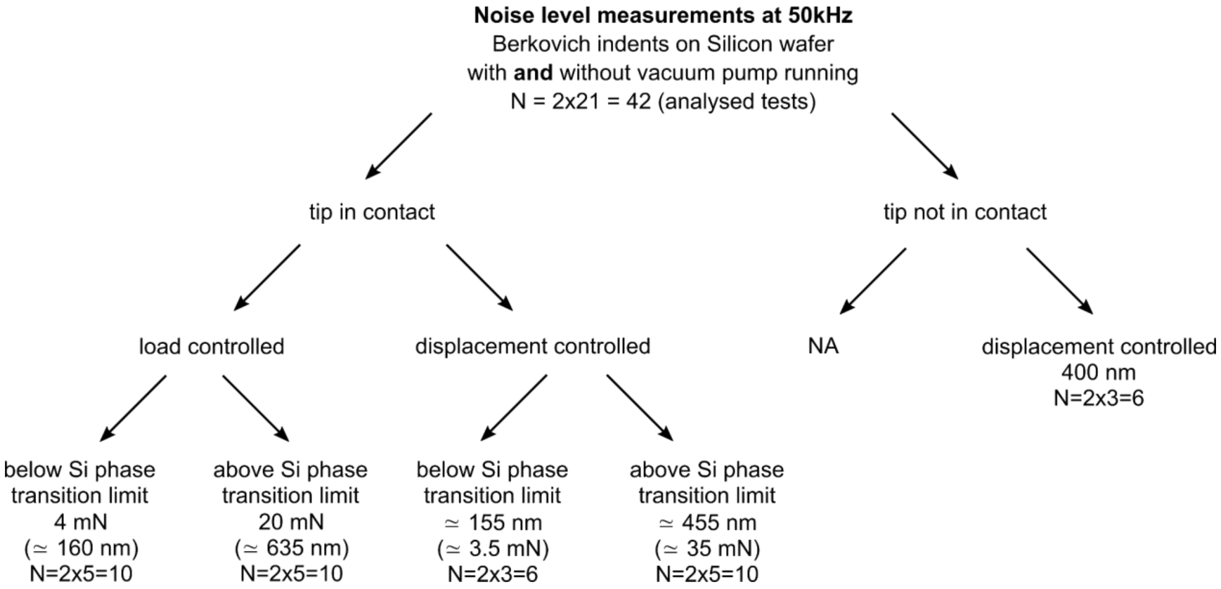

Figure S6. Measurement tree for the noise level tests for load- and displacement-controlled tests on a Si wafer.

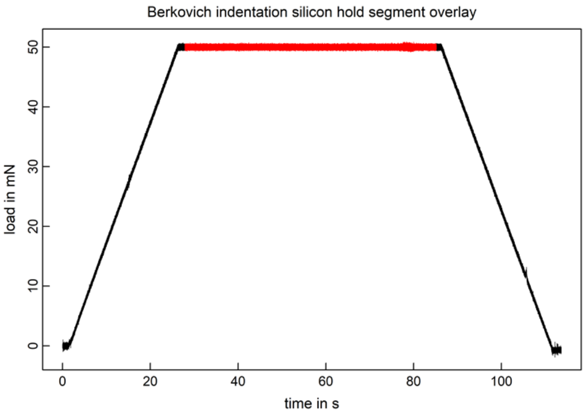

Figure S7. Exemplary load-controlled test to estimate the influence of the vacuum pump to hold the 4-inch wafer in place during the mapping experiments. Root mean square (RMS) and peak-to-peak noise levels of the red hold segment were analyzed.

Table S5: Test results of the noise measurements to determine the influence of the vacuum pump on load and displacement signals during Berkovich indentations. No significant differences (student t-test) between test with and without the vacuum pump running were detected with a significance level of p=0.05.

**Loading protocol**	**RMS noise/mN**	**Peak-Peak noise / mN**	**RMS noise / nm**	**Peak-Peak noise / nm**
4 mN no pump	0.097 ± 0.001	1.115 ± 0.107	9.473 ± 0.016	93.198 ± 1.736
4 mN with pump	0.098 ± 0.003	1.149 ± 0.247	9.454 ± 0.022	92.491 ± 2.056
20 mN no pump	0.105 ± 0.004	1.160 ± 0.184	9.502 ± 0.026	93.726 ± 3.425
20 mN with pump	0.109 ± 0.005	1.160 ± 0.165	9.456 ± 0.018	94.023 ± 3.385
155 mN no pump	0.097 ± 0.004	1.082 ± 0.049	9.455 ± 0.003	94.331 ± 1.770
155 mN with pump	0.103 ± 0.005	1.110 ± 0.030	9.454 ± 0.004	92.764 ± 5.854
455 mN no pump	0.148 ± 0.006	1.552 ± 0.042	9.457 ± 0.005	97.415 ± 7.008
455 mN with pump	0.151 ± 0.003	1.673 ± 0.099	9.458 ± 0.007	94.145 ± 1.929
Air no pump	0.096 ± 0.001	1.095 ± 0.043	9.454 ± 0.004	91.768 ± 1.810
Air with pump	0.096 ± 0.001	1.084 ± 0.057	9.458 ± 0.006	93.780 ± 3.007

Table S6. The features and their formulae used in the machine learning regression models.

Feature	Formula	Ref.
**δ_r**<br>**atomic size mismatch**	$\delta_r = \sqrt{\sum_{i=1}^{n} c_i \left(1 - \frac{r_i}{\bar{r}}\right)^2}$<br>$\bar{r} = \sum_{i=1}^{n} c_i r_i$<br>where n the number of the components in an alloy system, c_i the atomic percentage of the ith component, r_i atomic radius	[36]
**D.r**<br>**local atomic size mismatch**	$D.r = \sum_{i=1}^{n} \sum_{j=1, i \neq j}^{n} c_i c_j \lvert r_i - r_j \rvert$	[57]
**S_E/k_B**<br>**excess configurational entropy**	$\frac{S_E}{k_B} = \frac{(F - F^{id})}{k_B T} - lnZ - (3 - 2\xi)(1 - \xi)^{-2} + 3 + ln[(1 + \xi + \xi^2 - \xi^3)(1 - \xi)^{-3}]$<br>in which:<br>$\frac{(F - F^{id})}{k_B T} = -\frac{3}{2}(1 - y_1 + y_2 + y_3) + (3y_2 + 2y_3)(1 - \xi)^{-1} + \frac{3}{2}(1 - y_1 - y_2 - \frac{1}{3}y_3)(1 - \xi)^{-2} + (y_3 - 1)\ln(1 - \xi)$<br>The compressibility Z of hard spheres can be defined as:<br>$Z = [(1 + \xi + \xi^2) - 3\xi(y_1 + y_2\xi) - \xi^3 y_3](1 - \xi)^{-3}$<br>in which:<br>$y_1 = \sum_{j>i=1}^{m} \Delta_{ij}(d_i + d_j)(d_i d_j)^{-\frac{1}{2}}$<br>$y_2 = \sum_{j>i=1}^{m} \Delta_{ij} \sum_{k=1}^{m} \left(\frac{\xi_k}{\xi}\right) \frac{(d_i d_j)^{\frac{1}{2}}}{d_k}$<br>$y_3 = \left[\sum_{i=1}^{m} \left(\frac{\xi_i}{\xi}\right)^{1/2} c_i^{1/3}\right]$<br>$\Delta_{ij} = \left(\frac{(\xi_i \xi_j)^{1/2}}{\xi}\right)\left[\frac{(d_i - d_j)^2}{d_i d_j}\right](c_i c_j)^{1/2}$<br>$\xi = \sum_{i=1}^{m} \xi_i$<br>$\xi_i = \frac{1}{6}\pi\rho d_i^3 c_i$<br>where k_B is Boltzmann's constant, d_i is the atomic diameter of the ith element, c_i is the mole fraction of the ith element, ρ is the number density, which is temperature dependent, and ξ is the overall atomic packing fraction corresponding to a given ρ	[62,63]
**Λ**<br>**entropy and atomic size ratio**	$\Lambda = \frac{\Delta S_{mix}}{{\delta_r}^2}$<br>where ΔS_{mix} is the configuration entropy of mixing for an ideal solution ΔS_{mix}	[9,64]
**γ**<br>**the largest and the smallest atom**	$\gamma = \left(1 - \sqrt{\frac{(r_s + \bar{r})^2 - \bar{r}^2}{(r_s + \bar{r})^2}}\right) \Big/ \left(1 - \sqrt{\frac{(r_L + \bar{r})^2 - \bar{r}^2}{(r_L + \bar{r})^2}}\right)$	[9,65]
**ΔH_{mix}**<br>**enthalpy of mixing**	$\Delta H_{mix} = \sum_{i=1, i \neq j}^{n} \Omega_{ij} c_i c_j$<br>$\Omega_{ij} = 4\Delta H_{AB}^{mix}$<br>where Ω_{ij} is the regular solution interaction parameter between the ith and j^{th} elements, c_i and c_j are the atomic percentage of the ith and j^{th} elements, respectively, and ΔH_{AB}^{mix} is the enthalpy of mixing in an A–B system at an equiatomic composition, which can be determined based on Miedema's macroscopic model for binary alloys [72,73].	[63]
**Ω**<br>**competition between entropy and enthalpy of mixing in liquid phase**	$\Omega = \frac{T_m \Delta S_{mix}}{\lvert \Delta H_{mix} \rvert}$<br>$T_m = \sum_{i=1}^{n} c_i (T_m)_i$<br>where $(T_m)_i$ is the melting point of the ith element,	[9,68]
**ϕ**<br>**entropy effect gauge**	$\phi = \frac{\Delta S_{mix} - \frac{\lvert \Delta H_{mix} \rvert}{T_m}}{\lvert S_E \rvert}$	[9,67]
**Φ_f**<br>**competition between entropy and enthalpy of**	$\Phi_f = \frac{T_{ann} \Delta S_{mix}}{\lvert \Delta H_f \rvert}$<br>where T_{ann} is the annealing temperature used to achieve the corresponding equilibrium state during experiments, ΔH_f enthalpy of formation for the ordered binary compounds	[9,68]

**formation of binary compounds**		
**VEC_{LM}**<br>**local mismatch of valence electron concentration**	$$VEC_{LM} = \sum_{i=1}^{n} \sum_{j=1, i \neq j}^{n} c_i c_j \lvert VEC_i - VEC_j \rvert$$<br>where VEC_i is the valence electron concentration of the ith element	
**e/a_{LM}**<br>**local mismatch of itinerant electron concentration**	$$e/a_{LM} = \sum_{i=1}^{n} \sum_{j=1, i \neq j}^{n} c_i c_j \lvert e/a_i - e/a_j \rvert$$<br>where e/a_i is the itinerant electron concentration of the ith element	
**$\chi Allen_{LM}$**<br>**local mismatch of Allen's electronegativity**	$$\chi Allen_{LM} = \sum_{i=1}^{n} \sum_{j=1, i \neq j}^{n} c_i c_j \lvert \chi Allen_i - \chi Allen_j \rvert$$<br>where e/a_i is the itinerant electron concentration of the ith element	[69]
**η**<br>**modulus mismatch in strengthening model**	$$\eta = \sum_{i=1}^{n} \frac{c_i \frac{2(G_i - \bar{G})}{G_i + \bar{G}}}{1 + 0.5 \left\lvert c_i \frac{2(G_i - \bar{G})}{G_i + \bar{G}} \right\rvert}$$<br>where G_i is the shear modulus of the ith element	[58]
**A**<br>**energy term in strengthening model**	$$A = \frac{\bar{G}\delta_r(1+\mu)}{(1-\mu)}$$	[59]
**F**<br>**Peierls-Nabarro factor**	$$F = \frac{2\bar{G}}{1-\mu}$$	[60]
**w**<br>**six square of work function**	$$w = \left(\sum_{i=1}^{n} c_i w_i\right)^6$$<br>where w_i work function of i element	[61]
**G**<br>**shear modulus**	$$\bar{G} = \sum_{i=1}^{n} c_i G_i$$	[60]
**δG**<br>**shear modulus mismatch**	$$\delta G = \sqrt{\sum_{i=1}^{n} c_i \left(1 - \frac{G_i}{\bar{G}}\right)^2}$$	[60]
**D.G**<br>**local size shear modulus mismatch**	$$D.G = \sum_{i=1}^{n} \sum_{j=1, i \neq j}^{n} c_i c_j \lvert G_i - G_j \rvert$$	[60]
**μ**<br>**lattice distortion energy**	$$\mu = \frac{1}{2}\bar{E}\delta_r$$<br>where $\bar{E}$ is a mean value of Young's modulus	[60,71]

Table S7. The hyperparameter grid used in the last iteration to evaluate the regression models.

Model	Hyperparameter	Values
**MLP**	alpha	0.001, 0.005, 0.01, 0.05
	max_iter	2000, 5000, 10000
	hidden_layer_sizes	(4,), (6,), (8,), (10,), (12,), (14,), (16,), (20,),<br>(6, 6), (6, 4), (8, 8), (8, 6), (8, 4), (10, 10), (10, 8), (10, 6),
	activation	Identity, logistic, tanh, relu
	solver	lbfg, sgd, adam
**SVR**	C	0.1, 1, 2, 5, 10, 15, 50, 100
	gamma	0.001, 0.01, 0.1, 1, 10
	kernel	rbf, poly, linear
	degree	2, 3, 4, 5, 6,
**DT**	criterion	mse, mae
	min_samples_split	2, 5, 7, 10
	max_depth	2, 3, 5, 7, 9
	min_samples_leaf	2, 5, 10
	max_leaf_nodes	2, 5, 7, 10, 20, 30, 40, 50
**LGBM**	boosting_type	gbdt, dart, goss
	learning_rate	0.05, 0.1, 0.2, 0.5
	n_estimators	3, 5, 7, 10, 15, 20, 50
	max_depth	1, 2, 3
	num_leaves	2, 5, 10, 15
	min_child_samples	2, 5, 7
	min_child_weight	0.001, 0.01, 0.1
	subsample	0.1, 0.2, 0.3, 0.5, 0.7
**AB**	n_estimators	5, 10, 15, 20, 25, 30, 50
	learning_rate	0.01, 0.05, 0.1, 0.15, 0.5
**RF**	n_estimators	5, 10, 25, 50, 100
	max_depth	None, 2, 5, 7, 10 ,15
	min_samples_split	1, 2, 3, 5
	min_samples_leaf	1, 2, 3, 5
	bootstrap	True, False
**XGB**	learning_rate	0.05, 0.1, 0.15
	n_estimators	5, 10, 15, 50, 100, 200
	max_depth	2, 3, 5
	min_child_weight	1, 3, 5
	gamma	0.1, 0.5, 1
	subsample	0.4, 0.6, 0.8
**CB**	learning_rate	0.05, 0.1, 0.15
	n_estimators	40, 50, 60, 100, 200
	depth	2, 5, 7, 9
	l2_leaf_reg	0.5, 1, 2
	min_data_in_leaf	1, 3 , 5
	max_ctr_complexity	1, 2, 3
	Subsample	0.1, 0.5, 1.0
**KN**	n_neighbors	2, 3, 4, 5, 6
	leaf_size	2, 3, 5, 8, 10, 12, 15
	p	3, 5, 7, 9, 12, 15
